# Supplementary figures and images for: Loss of zebrafish atp6v1e1b, encoding a subunit of vacuolar ATPase, recapitulates human ARCL type 2C syndrome and identifies multiple pathobiological signatures
Source: PLoS Genet. 2021 Jun 18;17(6):e1009603. doi: 10.1371/journal.pgen.1009603 (PMC8244898; doi:10.1371/journal.pgen.1009603)

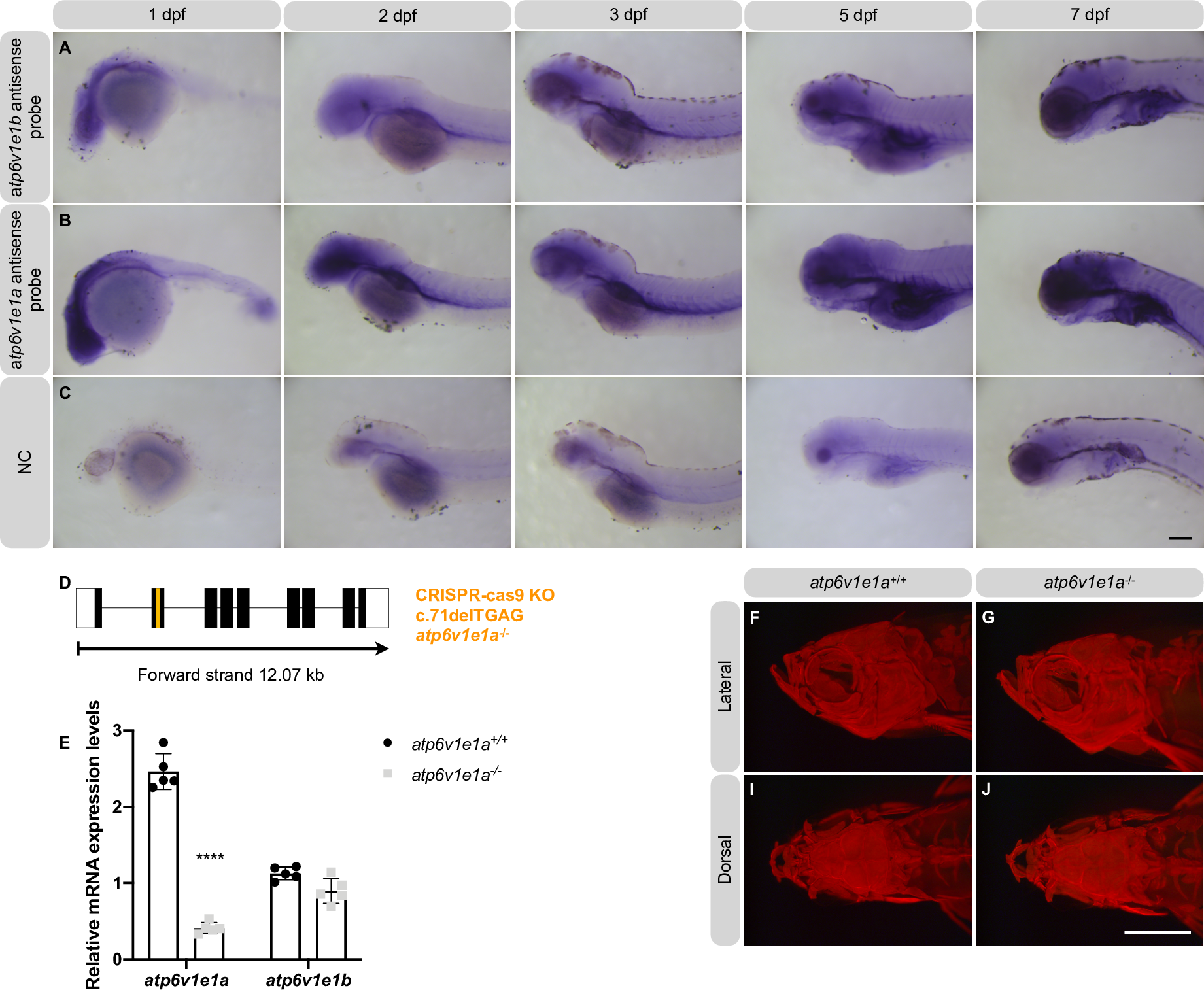

Supplement: S1 Fig — (A-C) Representative images demonstrating WISH for atp6v1e1a and atp6v1e1b at different developmental time points (24 hpf (n = 7), 48 hpf (n = 7), 72 hpf (n = 7), 120 hpf (n = 7) and 168 hpf (n = 7)). Expression patterns of atp6v1e1a and atp6v1e1b are similar. Scale bar = 200 μm. (D) Schematic representation of the atp6v1e1a gene (transcript ENSDART00000008986.6). The orange line represents the position of the target site of the sgRNA used for CRISPR-Cas9-induced indel mutagenesis (atp6v1e1a-/-). (E) RT-qPCR analysis showed a significant decrease in expression of atp6v1e1a at 3 dpf in atp6v1e1a-/-. Gene expression levels of atp6v1e1b remained normal. Data are expressed as mean ± SD from 5 biological replicates. 2-way ANOVA with Tukey test for multiple comparison. (F-J) Whole-mount bone staining of 12-month-old adult atp6v1e1a-/- and their respective wild-type (WT) controls in dorsal (I, J) and lateral positions (F, G). N = 8 per genotype. Scale bar = 2 mm (A, B, C, D). (TIF) [file pgen.1009603.s001.tif]

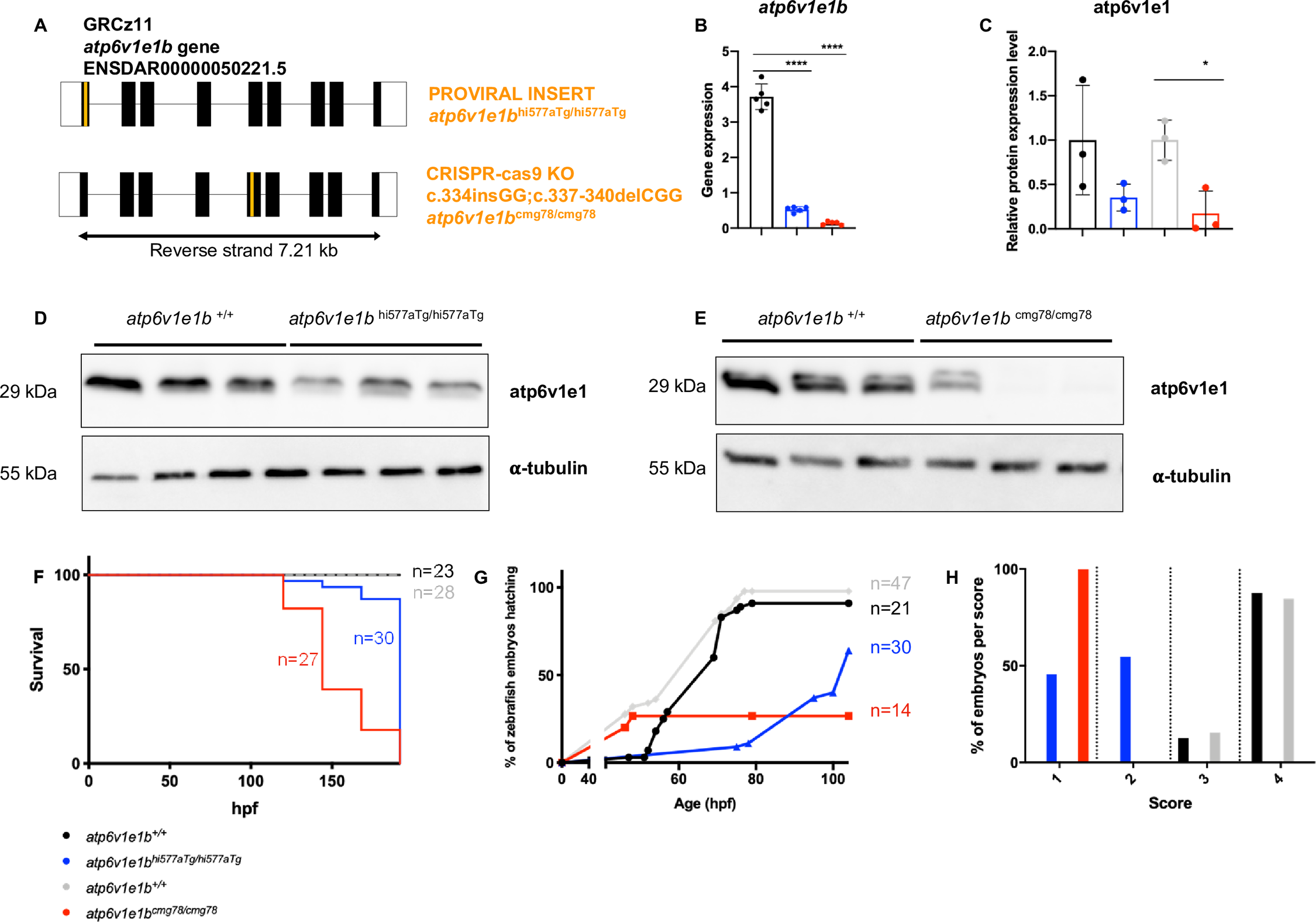

Supplement: S2 Fig — (A) Schematic representation of the atp6v1e1b gene (transcript ENSDAR00000050221.5). The orange line represents the position of the proviral insert (atp6v1e1bhi577aTg/+) or the target site of the sgRNA used for CRISPR-Cas9-induced indel mutagenesis (atp6v1e1bcmg78/+). (B) RT-qPCR analysis showed a significant decrease in expression of atp6v1e1b in both zebrafish models at 3 dpf. Data are expressed as mean ± SD from 5 biological replicates. (C) Band intensities of fluorescence signals of atp6v1e1 and α-tubulin were quantified and normalized to the corresponding WT controls. Data are expressed as mean ± SD from 3 biological replicates, in which 20 zebrafish larvae were pooled per sample. 2-way ANOVA with Tukey test for multiple comparison was used for statistical analysis. Different protein extracts were loaded on the immunoblot. (D-E) Immunoblot of lysates obtained from atp6v1e1bhi577aTg/hi577aTg, atp6v1e1bcmg78/cmg78, and their WT controls at 4 dpf. We confirmed equal loading by staining for α-tubulin. (F) Loss of atp6v1e1b causes embryonic mortality in zebrafish. Kaplan-Meier curves for survival of atp6v1e1b-deficient zebrafish. (G) Hatching pattern of atp6v1e1b-deficient zebrafish. Atp6v1e1b-defcient zebrafish showed delayed or no spontaneous hatching (H) Touch-Evoked Escape test (TEE-test) of atp6v1e1bhi577aTg/hi577aTg larvae (n = 22) and their respective WT controls (n = 8), atp6v1e1bcmg78/cmg78 (n = 26) larvae and their respective WT controls (n = 26). The reaction after touch with a thick blunt needle was scored as follows: (1) no movement, (2) local muscle contractions of the embryo, (3) short distance swim movement and (4) normal swim movement towards the edge of the Petri dish. (TIF) [file pgen.1009603.s002.tif]

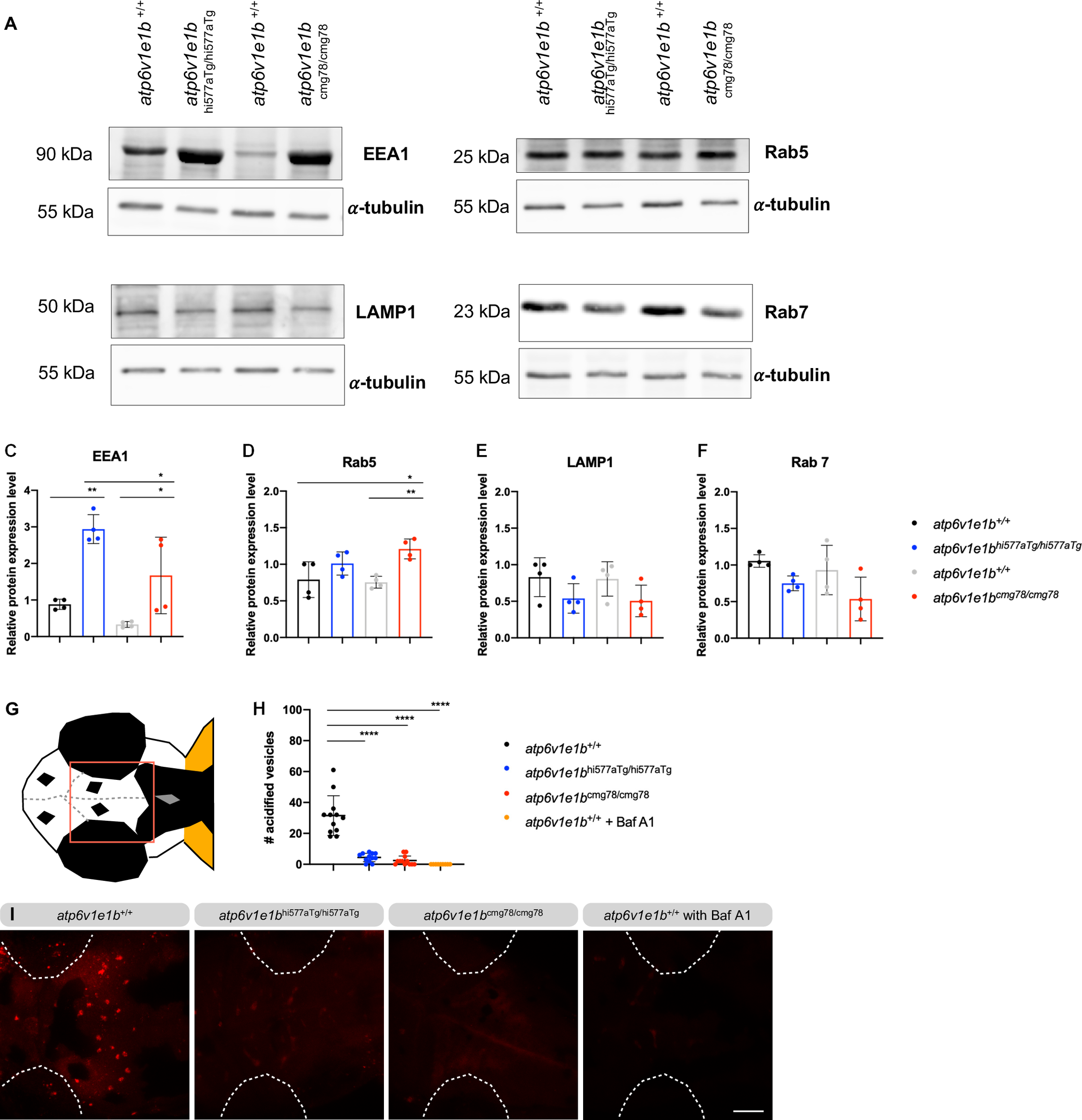

Supplement: S3 Fig — (A) Immunoblot of lysates from whole atp6v1e1bhi577aTg/hi577aTg, atp6v1e1bcmg78/cmg78, and corresponding WT control larvae at 4 dpf. We confirmed equal loading by staining for α-tubulin. (C-F) Quantification of the relative protein levels of EEA1 (marker for early endosomes), Rab5 (marker for early endosomes), Rab7 (marker for late endosomes), and LAMP1 (marker for lysosomes), normalized to total α-tubulin levels. Data are expressed as mean ± SD from 4 biological replicates. (G) Schematic presentation of the area covered by confocal microscopy in I. (H) The number of acidified vesicles is significantly reduced in atp6v1e1bhi577aTg/hi577aTg, atp6v1e1bcmg78/cmg78, and Baf A1 treated WT larvae at 3 dpf compared to WT controls. (I) Representative confocal images of the cranial area are shown. Outlines of the eyes are indicated by dotted lines. Acidified vesicles in brain cells are visualized in red, including lysosomes, endosomes and exosomes. Atp6v1e1b+/+ (n = 12), atp6v1e1bhi577aTg/hi577aTg (n = 12), atp6v1e1bcmg78/cmg78 (n = 12) and atp6v1e1b+/+ treated with 1.6 μM Baf A1 (n = 9). Scale bar = 100 μm. (TIF) [file pgen.1009603.s003.tif]

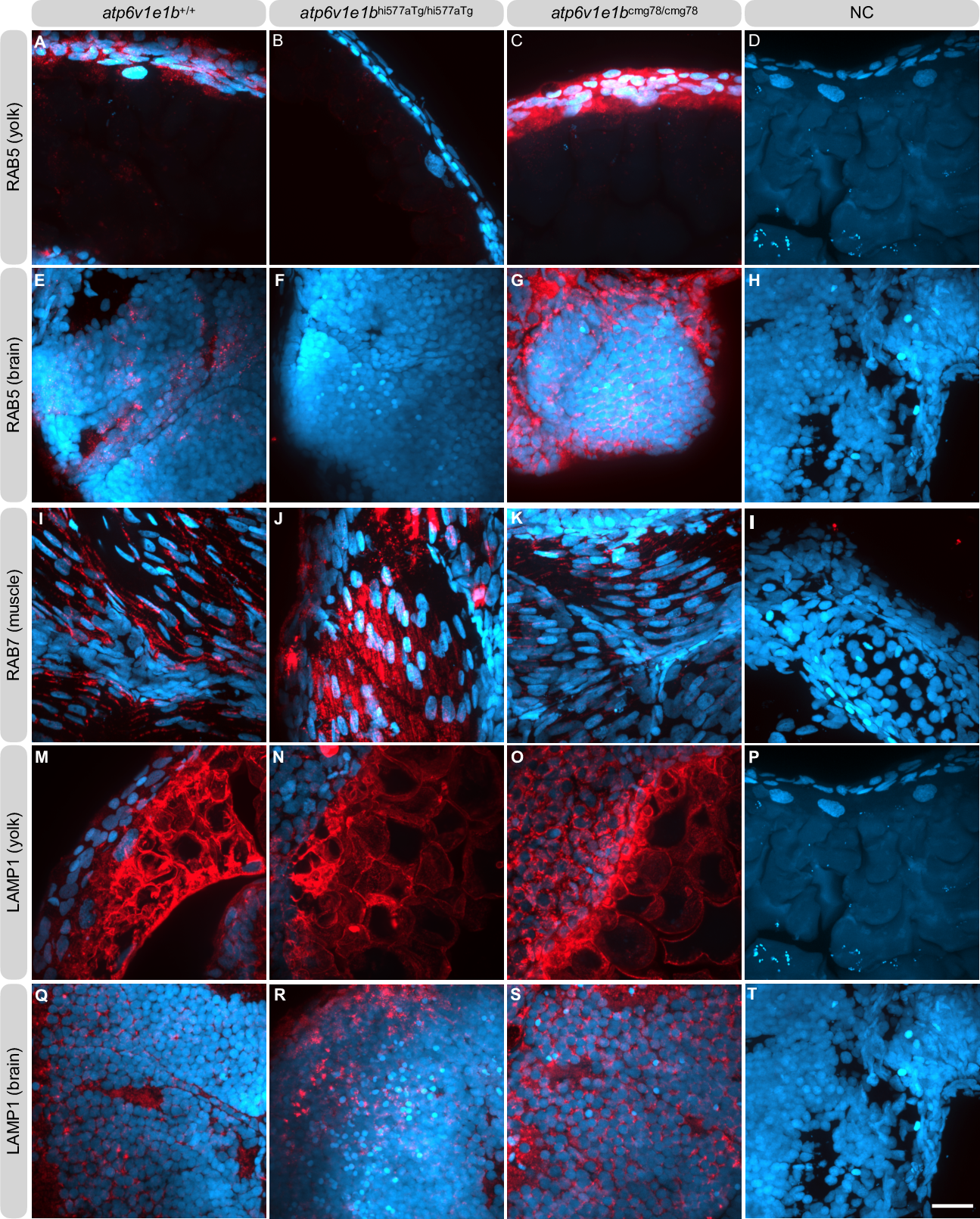

Supplement: S4 Fig — Representative images are shown. Confocal microscopy images of longitudinal sections of the yolk (A-D) and brain (E-H) from 4 dpf atp6v1e1b+/+, atp6v1e1bhi577aTg/hi577aTg, and atp6v1e1bcmg78/cmg78 larvae stained for RAB5. (I-L) Confocal microscopy images of longitudinal sections of the muscle from the trunk of the zebrafish larvae from 3 dpf atp6v1e1b+/+, atp6v1e1bhi577aTg/hi577aTg, and atp6v1e1bcmg78/cmg78 larvae stained for RAB7. Confocal microscopy images of longitudinal sections of the yolk (M-P) and brain (Q-T) from 3 dpf atp6v1e1b+/+, atp6v1e1bhi577aTg/hi577aTg, and atp6v1e1bcmg78/cmg78 larvae stained for LAMP1. Negative control was imaged for each corresponding region in the zebrafish larvae. Scale bar = 20 μm. (TIF) [file pgen.1009603.s004.tif]

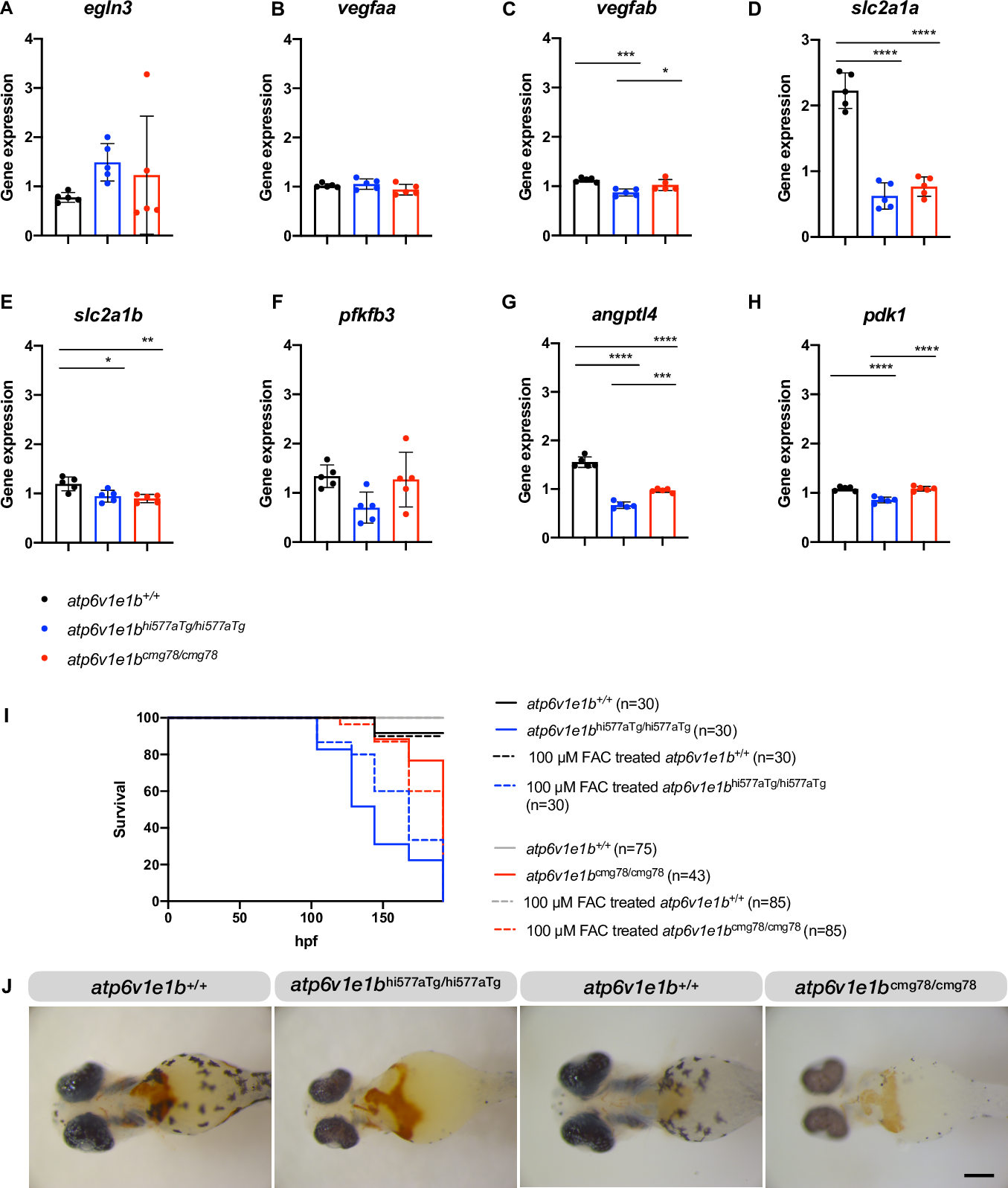

Supplement: S5 Fig — (A-H) No difference in gene expression of the HIF-1α-target genes, egln3, vegfaa and pfkfb3, were found in atp6v1e1b-deficient zebrafish. Interestingly, the gene expression of the HIF-1α-target genes, vegfab, slc2a1a, slc2a1b, angptl4 and pdk1, was downregulated to varying extents in atp6v1e1b-deficient zebrafish based on RT-qPCR. Data are expressed as mean ± SD from 5 biological replicates. (I) Supplementation of 100 μM iron (Fe3+) ammonium citrate (FAC) does not improve survival of atp6v1e1b-deficient zebrafish. Kaplan-Meier curves for survival of atp6v1e1b-deficient zebrafish with and without FAC treatment. (J) Atp6v1e1b-deficient and WT control zebrafish at 3 dpf exhibited normal levels of hemoglobin upon o-dianisidine staining. Atp6v1e1bhi577aTg/hi577aTg (n = 15) and their respective WT controls (n = 15), atp6v1e1bcmg78/cmg78 (n = 15) and their respective WT controls (n = 15). Scale bar: 200 μM. (TIF) [file pgen.1009603.s005.tif]

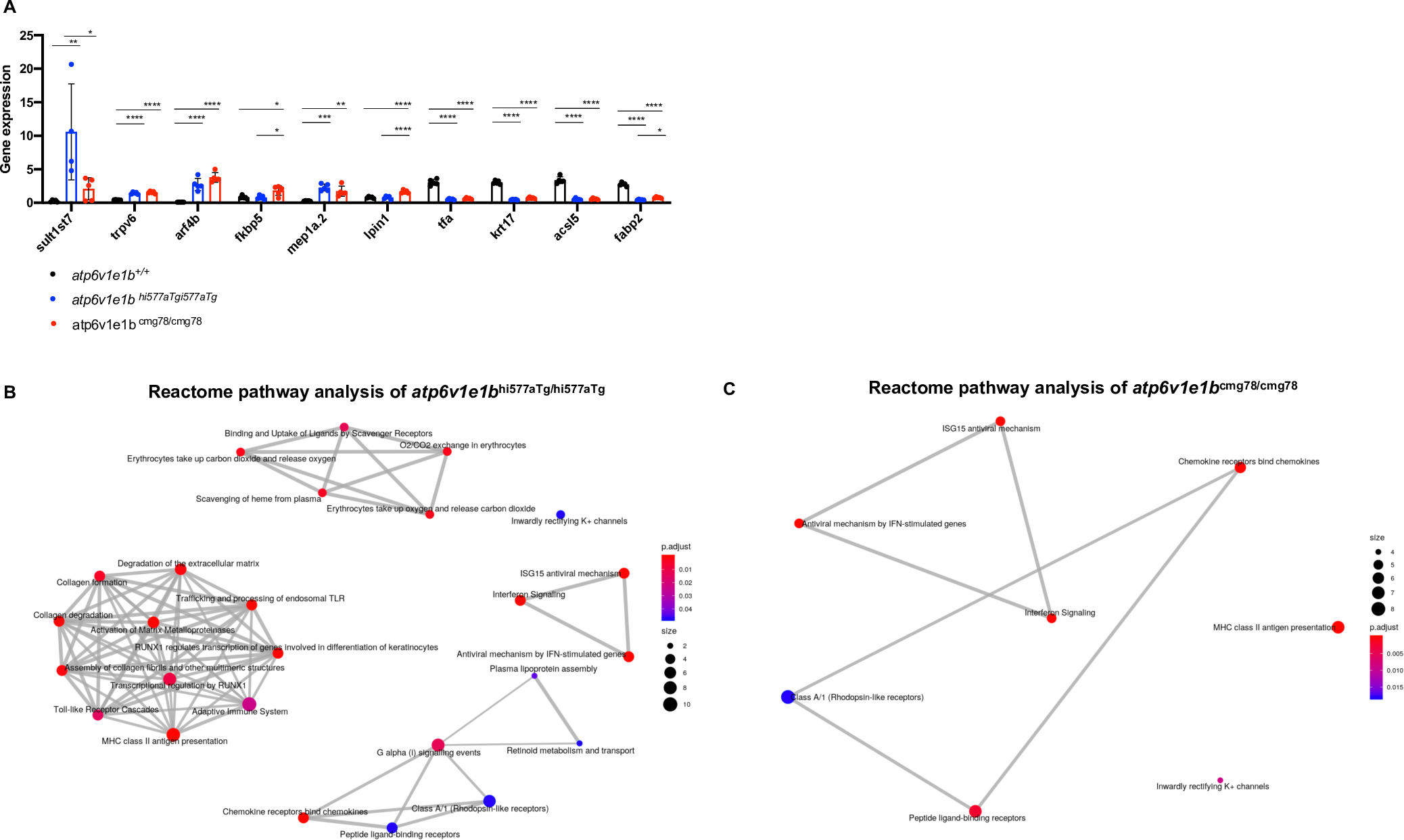

Supplement: S6 Fig — (A) RT-qPCR analysis of 10 from the top150 DEGs validating the results obtained in the unbiased transcriptomic analysis. Data are expressed as mean ± SD from 5 biological replicates. (B-C) Cluster plot showing the top enriched reactome pathways of the DEG from whole-body samples of atp6v1e1b-deficient and WT control zebrafish at 3 dpf. (TIF) [file pgen.1009603.s006.tif]

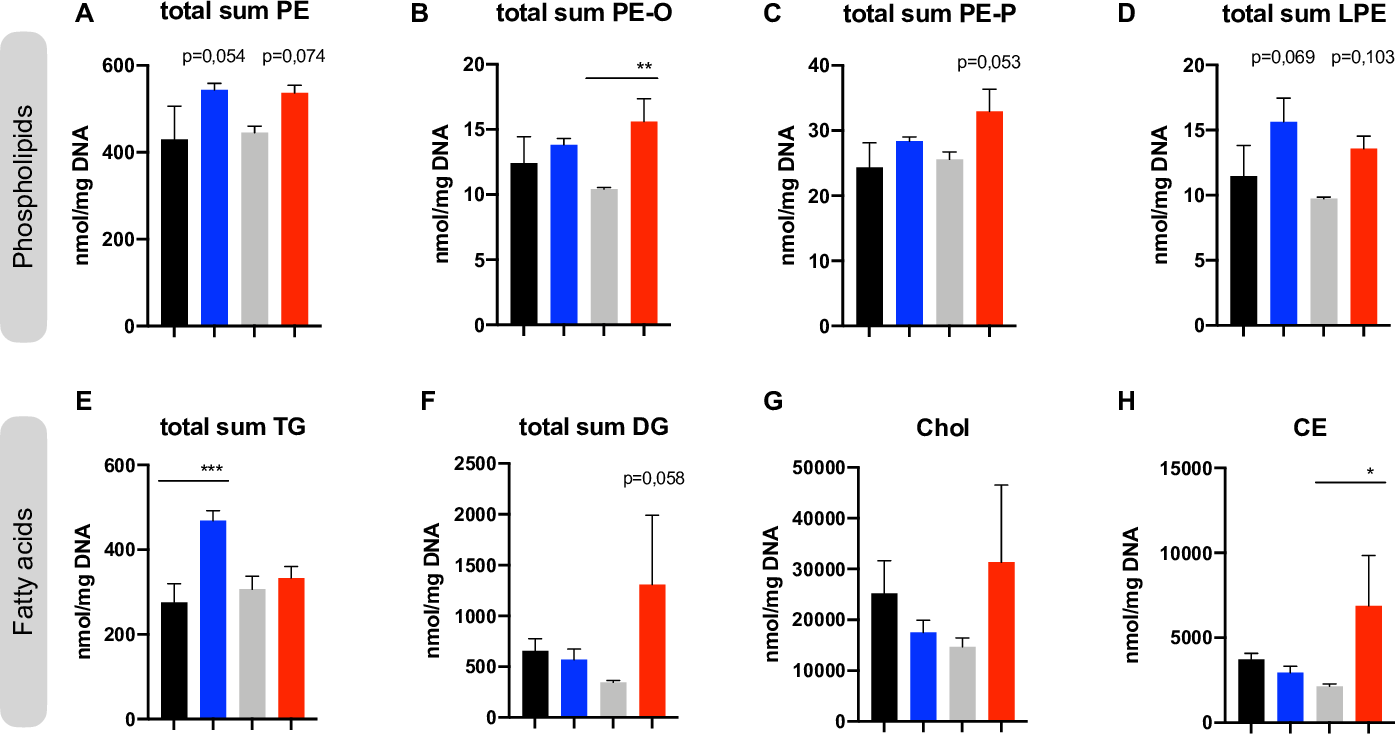

Supplement: S7 Fig — (A-H) HILIC LC MS/MS lipidomic analysis demonstrates minor changes in the total levels of phospholipids and fatty acids in 3 dpf atp6v1e1b-deficient zebrafish. Data are expressed as mean ± SD from 3 biological replicates. TG: triacylglycerides; DG: diacylglycerides; Chol: cholesterol; CE: cholesterol esters; PE: phosphatidylethanolamine; PE-O: 1-alkyl,2- acylphosphatidylethanolamines; PE-P: 1-alkenyl,2-acylphosphatidylethanolamines; LPE: lysophosphatidylethanolamine. (TIF) [file pgen.1009603.s007.tif]

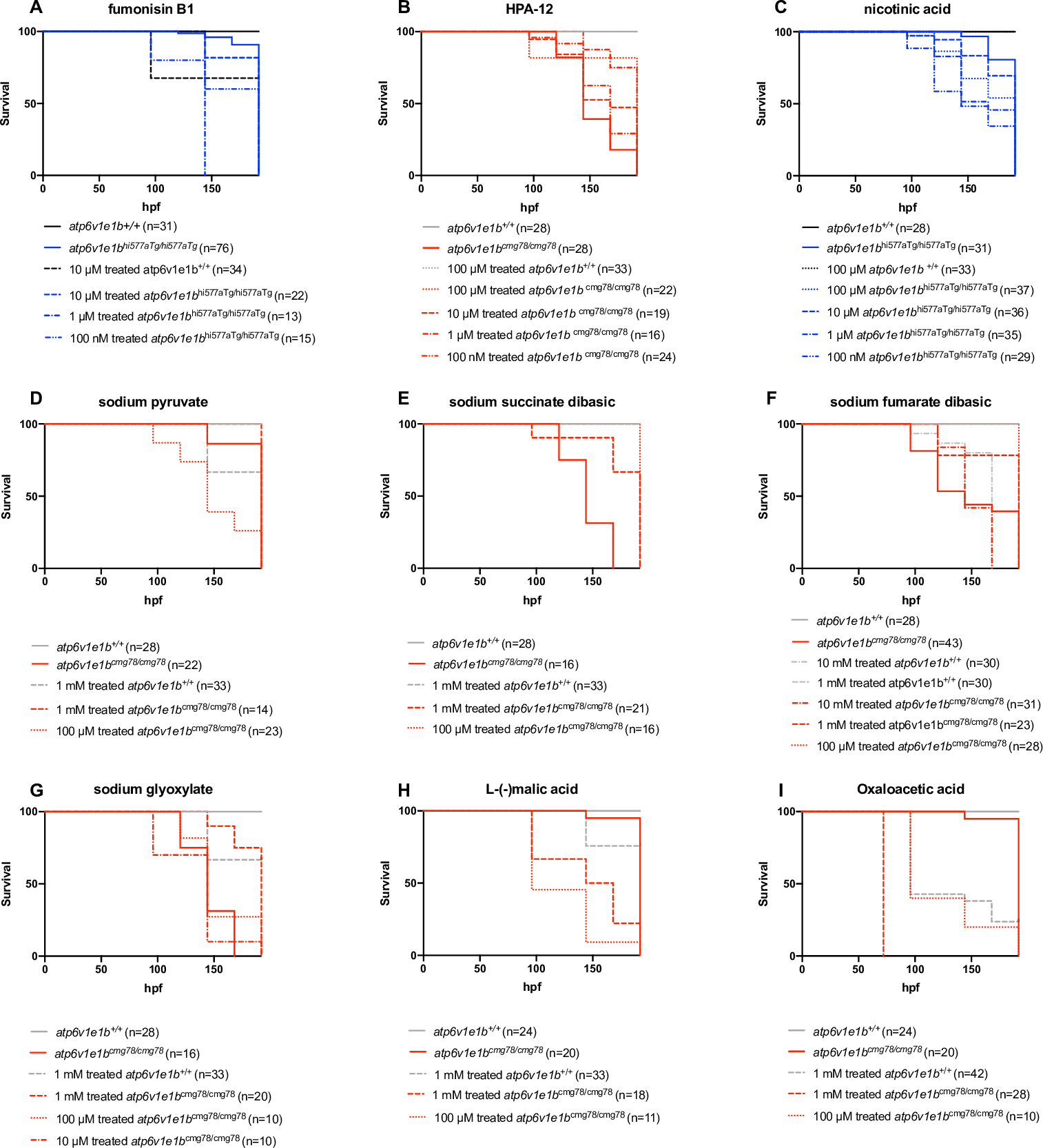

Supplement: S8 Fig — (A-I) Survival curves are shown for atp6v1e1b-deficient zebrafish and controls until 8 dpf. Compounds were administered at 1 dpf, after chorion removal. (A-C) Administration of compounds interfering with the ceramide biosynthesis and cholesterol esterification. (D-I) Administration of compounds influencing the Krebs cycle. Kaplan-Meier curves for survival of atp6v1e1b-deficient and treated atp6v1e1b-deficient zebrafish. (TIF) [file pgen.1009603.s008.tif]

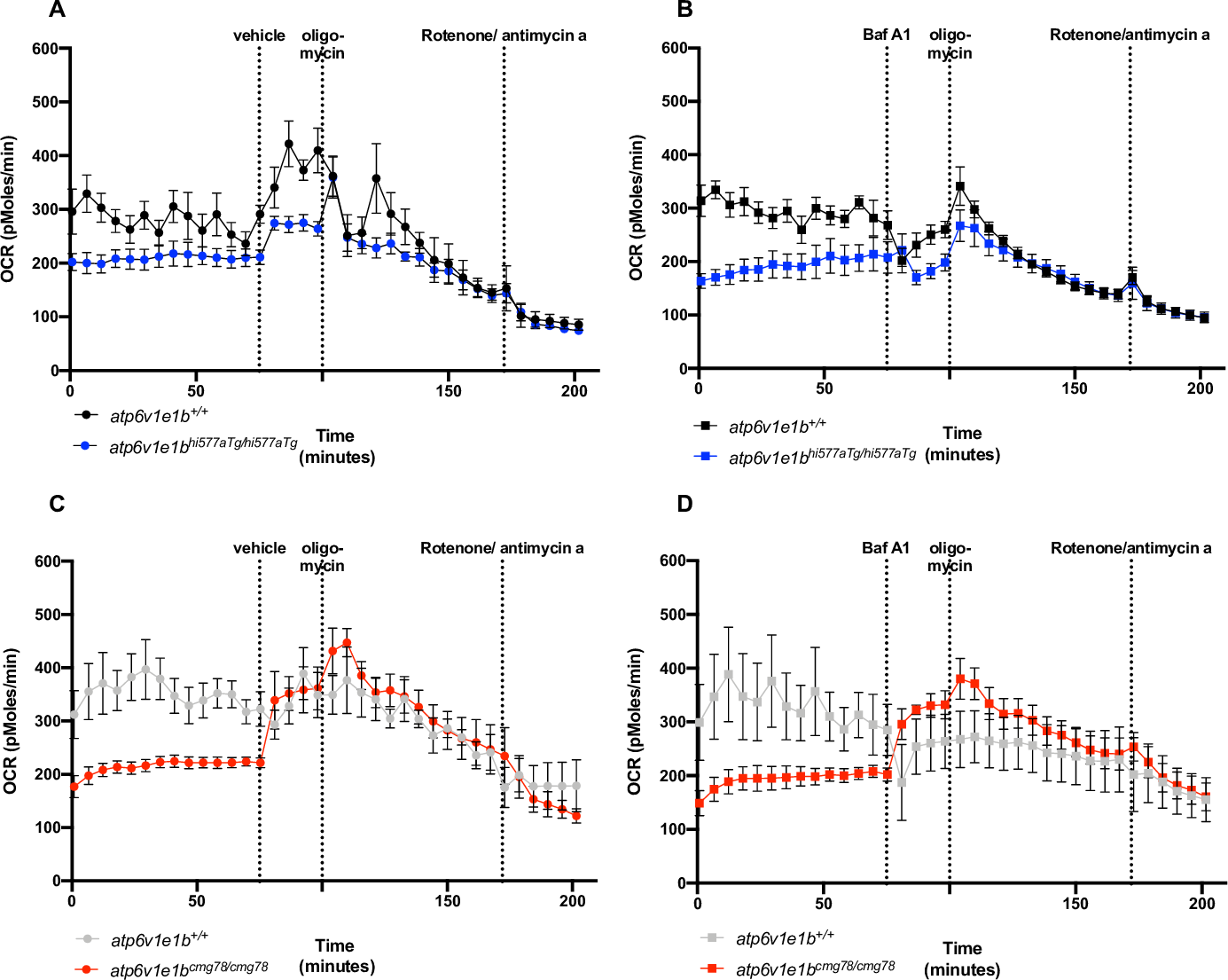

Supplement: S9 Fig — OCR response to oligomycin and rotenone/antimycin a in 2 dpf atp6v1e1bhi577aTg/hi577aTg (A-B) and atp6v1e1bcmg78/cmg78 zebrafish (C-D) and their respective controls after administration of vehicle (A, C) or the v-ATPase inhibitor Baf A1 (B, D) with oligomycin and rotenone/antimycin a or after administration. (C-D) OCR in WT controls and atp6v1e1bcmg78/cmg78 zebrafish after administration of vehicle in C or Baf A1 in D with oligomycin and rotenone/antimycin a or after administration. Data are expressed as mean ± SEM from 5 biological replicates in which 4 zebrafish were pooled. Baf A1: Bafilomycin A1; OCR: oxygen consumption rate. (TIF) [file pgen.1009603.s009.tif]
