# Supplementary material for: Loss of zebrafish atp6v1e1b, encoding a subunit of vacuolar ATPase, recapitulates human ARCL type 2C syndrome and identifies multiple pathobiological signatures
Source: PLoS Genet. 2021 Jun 18;17(6):e1009603. doi: 10.1371/journal.pgen.1009603 (PMC8244898; doi:10.1371/journal.pgen.1009603)
Supplement: S1 Table — (DOCX) [file pgen.1009603.s010.docx]

**S1 Table: List of DEGs with unknown gene names.**

| Ensemble | ZFIN | Chromosome | Predicted gene association |
| --- | --- | --- | --- |
| CU984600.2 | si:dkeyp-118h9.7 | Chromosome 9: 563,547-582,037 forward strand | GTPBP2 (HGNC Symbol) |
| si:ch1073-340i21.3 | si:ch1073-340i21.3 | Chromosome 15: 46,329,149-46,336,399 forward strand. | *fb16f09* |
| si:dkey-35h6.1 | si:dkey-35h6.1 | Chromosome 14: 13,036,560-13,048,355 reverse strand. | NA |
