# Supplementary material for: Loss of zebrafish atp6v1e1b, encoding a subunit of vacuolar ATPase, recapitulates human ARCL type 2C syndrome and identifies multiple pathobiological signatures
Source: PLoS Genet. 2021 Jun 18;17(6):e1009603. doi: 10.1371/journal.pgen.1009603 (PMC8244898; doi:10.1371/journal.pgen.1009603)
Supplement: S2 Table — (Left) GSEA algorithm which considers the overall ranks of features without using a significance cut-off. (Right) Mummichog algorithm which implements an over-representation analysis method that is not able to detect subtle changes. *The mummichog compound hits represent the number of significant compounds divided by the total number of compounds per pathway. (DOCX) [file pgen.1009603.s011.docx]

**S2 Table: Pathway activity profile of metabolites from *atp6v1e1b*-deficient zebrafish.**

| GSEA  *atp6v1e1b*^+/+^ vs a*tp6v1e1b*^hi577aTg/hi55aTg^ | | | Mummichog  *atp6v1e1b*^+/+^ vs a*tp6v1e1b*^hi577aTg/hi55aTg^ | | |
| --- | --- | --- | --- | --- | --- |
| Pathway Name | **Compound Hits** | **p-value** | **Pathway Name** | **Compound Hits*** | **p-value** |
| Butanoate metabolism | 1 | 0.01786 | Glycolysis and Gluconeogenesis | 5/53 | 0.018478 |
| Fatty acid activation | 1 | 0.01786 | Butanoate metabolism | 1/31 | 0.13636 |
| Fatty acid oxidation | 1 | 0.01786 | Fatty acid activation | 1/38 | 0.13636 |
| Fatty acid oxidation- peroxisome | 1 | 0.01786 | Fatty acid oxidation | 1/78 | 0.13636 |
| Propanoate metabolism | 1 | 0.01786 | Fatty acid oxidation- peroxisome | 1/29 | 0.13636 |
| Heparan sulfate degradation | 2 | 0.01887 | Propanoate metabolism | 1/25 | 0.13636 |
| De novo fatty acid biosynthesis | 2 | 0.02 | Starch and sucrose metabolism | 1/2 | 0.13636 |
| Prostaglandin formation from arachidonate | 2 | 0.02 | Valine- leucine and isoleucine degradation | 5/66 | 0.13755 |
| Squalene and cholesterol biosynthesis | 2 | 0.02 | Aspartate and asparagine metabolism | 12/84 | 0.21253 |
| Vitamin E metabolism | 2 | 0.02 | TCA cycle | 7/37 | 0.24383 |
| Glycerophospholipid metabolism | 17 | 0.02174 | Xenobiotics metabolism | 7/103 | 0.24383 |
| Starch and sucrose metabolism | 1 | 0.02174 | De novo fatty acid biosynthesis | 2/73 | 0.2549 |
| Tryptophan metabolism | 7 | 0.02222 | Leukotriene metabolism | 2/89 | 0.2549 |
| Tyrosine metabolism | 16 | 0.025 | Prostaglandin formation from arachidonate | 2/48 | 0.2549 |
| Glycolysis and Gluconeogenesis | 5 | 0.04 | Squalene and cholesterol biosynthesis | 2/53 | 0.2549 |
| Leukotriene metabolism | 2 | 0.04 | Vitamin B1 (thiamin) metabolism | 2/22 | 0.2549 |
| Ubiquinone Biosynthesis | 5 | 0.04 | Vitamin E metabolism | 2/26 | 0.2549 |
| Vitamin B1 (thiamin) metabolism | 2 | 0.04 |  |  |  |
| Fructose and mannose metabolism | 3 | 0.04444 |  |  |  |
| Hexose phosphorylation | 3 | 0.04444 |  |  |  |
| Butanoate metabolism | 1 | 0.01786 |  |  |  |
| Fatty acid activation | 1 | 0.01786 |  |  |  |
| Fatty acid oxidation | 1 | 0.01786 |  |  |  |
| Fatty acid oxidation- peroxisome | 1 | 0.01786 |  |  |  |
| Propanoate metabolism | 1 | 0.01786 |  |  |  |
| Heparan sulfate degradation | 2 | 0.01887 |  |  |  |
| De novo fatty acid biosynthesis | 2 | 0.02 |  |  |  |
| Prostaglandin formation from arachidonate | 2 | 0.02 |  |  |  |

| GSEA  *atp6v1e1b*^+/+^ vs a*tp6v1e1b*^cmg78/cmg78^ | | | Mummichog  *atp6v1e1b*^+/+^ vs a*tp6v1e1b*^cmg78/cmg78^ | | |
| --- | --- | --- | --- | --- | --- |
| Pathway Name | **Compound Hits** | **p-value** | **Pathway Name** | **Compound Hits** | **p-value** |
| Sialic acid metabolism | 10 | 0.01587 | De novo fatty acid biosynthesis | 2/73 | 0.027914 |
| Starch and sucrose metabolism | 1 | 0.01786 | TCA cycle | 8/37 | 0.029255 |
| De novo fatty acid biosynthesis | 2 | 0.02222 | Aspartate and asparagine metabolism | 13/84 | 0.047983 |
| Prostaglandin formation from arachidonate | 3 | 0.02222 | Glycolysis and Gluconeogenesis | 3/7 | 0.095707 |
| Squalene and cholesterol biosynthesis | 2 | 0.02222 | Glutathione Metabolism | 2/4 | 0.13372 |
| Tryptophan metabolism | 8 | 0.02439 | Valine- leucine and isoleucine degradation | 3/8 | 0.13558 |
| Glycosphingolipid metabolism | 7 | 0.025 | Butanoate metabolism | 1/1 | 0.16923 |
| Glycerophospholipid metabolism | 16 | 0.02632 | Fatty acid activation | 1/1 | 0.16923 |
| Purine metabolism | 18 | 0.02632 | Fatty acid oxidation | 1/1 | 0.16923 |
| Tyrosine metabolism | 23 | 0.02857 | Fatty acid oxidation- peroxisome | 1/1 | 0.16923 |
| Chondroitin sulfate degradation | 3 | 0.03448 | Keratan sulfate biosynthesis | 1/1 | 0.16923 |
| Butanoate metabolism | 1 | 0.04348 | Propanoate metabolism | 1/1 | 0.16923 |
| Fatty acid activation | 1 | 0.04348 | Proteoglycan biosynthesis | 1/1 | 0.16923 |
| Fatty acid oxidation | 1 | 0.04348 | Starch and sucrose metabolism | 1/1 | 0.16923 |
| Fatty acid oxidation- peroxisome | 1 | 0.04348 | Glycine- serine- alanine and threonine metabolism | 3/10 | 0.22834 |
| Propanoate metabolism | 1 | 0.04348 |  |  |  |
| Vitamin B5 - CoA biosynthesis from pantothenate | 2 | 0.04444 |  |  |  |
| Vitamin E metabolism | 2 | 0.04444 |  |  |  |
| Methionine and cysteine metabolism | 14 | 0.04762 |  |  |  |
