# Supplementary material for: Loss of zebrafish atp6v1e1b, encoding a subunit of vacuolar ATPase, recapitulates human ARCL type 2C syndrome and identifies multiple pathobiological signatures
Source: PLoS Genet. 2021 Jun 18;17(6):e1009603. doi: 10.1371/journal.pgen.1009603 (PMC8244898; doi:10.1371/journal.pgen.1009603)
Supplement: S3 Table — (DOCX) [file pgen.1009603.s012.docx]

**S3 Table: Zebrafish mutation and primers used for genotyping.**

| Zebrafish line | Mutation | Forward primer | Reverse primer |
| --- | --- | --- | --- |
| *atp6v1e1b*^hi577aTg/+^ | Proviral insertion in 5’UTR | Proviral DNA: CGTCAGAACATCACGACACTCC | CATCCGACTTGTGGTCTCGC |
|  |  | Genomic DNA: CACGGCTCTGACACTGTCA |  |
| *atp6v1e1b*^cmg78/+^ | c.334insGG; c.337- 340delCGG | TCAAATGTCCAACCTGATGA | CCAACAAACGCCCTGTAATA |
| *atp6v1e1a*^cmg74/+^ | c.69delCCAGTGAGAAG | CATCCTGCATTTTCATCACG | AGCCACTGCCAATACAACAA |
