# Supplementary material for: Loss of zebrafish atp6v1e1b, encoding a subunit of vacuolar ATPase, recapitulates human ARCL type 2C syndrome and identifies multiple pathobiological signatures
Source: PLoS Genet. 2021 Jun 18;17(6):e1009603. doi: 10.1371/journal.pgen.1009603 (PMC8244898; doi:10.1371/journal.pgen.1009603)
Supplement: S4 Table — (DOCX) [file pgen.1009603.s013.docx]

**S4 Table: Compound screen in zebrafish.**

| Compound | Supplier | Cat. No. |
| --- | --- | --- |
| Sodium fumarate dibasic | Sigma-Aldrich | F1506 |
| Sodium pyruvate | Sigma-Aldrich | P2256 |
| Oxaloacetic acid | Sigma-Aldrich | O4126 |
| L-(−)-Malic acid | Sigma-Aldrich | M1000 |
| Sodium succinate dibasic hexahydrate | Sigma-Aldrich | F1506 |
| Sodium glyoxylate monohydrate | Sigma-Aldrich | G4502 |
| Fumonisin B1 | R&D Systems Europe | 3103/1 |
| HPA-12 | TCI Europe NV | H1553 |
| Nicotinic acid | Sigma-Aldrich | N0761 |
| Ammonium iron(III) citrate | Sigma-Aldrich | F5879 |
