# Supplementary material for: Loss of zebrafish atp6v1e1b, encoding a subunit of vacuolar ATPase, recapitulates human ARCL type 2C syndrome and identifies multiple pathobiological signatures
Source: PLoS Genet. 2021 Jun 18;17(6):e1009603. doi: 10.1371/journal.pgen.1009603 (PMC8244898; doi:10.1371/journal.pgen.1009603)
Supplement: S5 Table — (DOCX) [file pgen.1009603.s014.docx]

**S5 Table: Primer sequences for qPCR analysis.**

| Gene Symbol | Forward primer sequence | Reverse primer sequence |
| --- | --- | --- |
| *col1a1a* | CGTAATGTGCGACGAAGTGA | CTAGGTCCCTCAACACTGGG |
| *col1a1b* | ATACAGAGTCAGCGAGGACG | CTACGCCGAATTCCTGATCG |
| *col1a2* | GCCTGGTAACATTGGATTCCC | GGGCTGCCTCTCAGTCC |
| *elnb* | AACCCGTGGTACTGGAGAAG | GTAACACCTGATCCCTCTACTGG |
| *elfa* | GGAGACTGGTGTCCTCAA | GGTGCATCTCAACAGACTT |
| *bactin2* | ACGATGGATGGGAAGACA | AAATTGCCGCACTGGTT |
| *gapdh* | GTGGAGTCTACTGGTGTCTTC | GTGCAGGAGGCATTGCTTACA |
| *egln3* | TGGGTTAATGGGACCGAGAG | TCCAGGATAACAAGCCACCAT |
| *vegfaa* | GAAACGTCACTATGGAGGTGC | GCTCACAGTGGTTTTCTTTCTTTG |
| *vegfab* | CTTCGTCATCAGGGTGACATT | AAGGCTCACACTGACTATTTTCTAT |
| *slc2a1a* | CATTAACGCTCCACAGAAGATCA | TCGGCGGAATGTACTCTGAA |
| *slc2a1b* | ATCCTCATGGCACAGGTCTTC | GGCAGTAAACAGCACTGTACC |
| *pfkfp3* | TGTCCACACAGAACTGGCT | CTGAAAGAGCAGGACTGACAC |
| *angptl4* | GAGCACTGAACAGCGAGATTC | AAAAGGCTGGGAGTCACTTG |
| *pdk1* | TGCTGTTATTTACATCCGAGCTTT | AGCCTCGTGGATGGTCTTAT |
| *sult1st7* | GGAGTCTTGTTTTCCAGGGATG | GAGTTTTGATTAGGCGAGGAGAAT |
| *trpv6* | ACTGCTTGACTGTTCATCTACTG | CAGATGTCATGGGCTCGTTG |
| *arf4b* | GAGGGGATTTGCTGTGTTTTCT | GAGGAAGACAGTGGTTTGTTCA |
| *fkbp5* | CGCTACGCACAGGTTGTATT | ATACATGCCCTTGTTCCCAAAA |
| *mepta.2* | TGATCTGGAGAGACTCAATCGC | TGATCTGCTCAAAGGCACACT |
| *tfa* | AGGAAAGAAGTCCTGCCACA | GCAGCCTTCGCTAAAGAACT |
| *krt17* | AACTCAGCATGAAAGCGTCA | ATCACCACGAAGATGCACCA |
| *lpin1* | ATGAAGCGTGAGGACAATGGA | ATGTGTTGCTTGCTCCTTTCAG |
| *acsl5* | GCAGTGCCTCTTTACGACAC | TCCAGAAGTGTTTCTGCTTTATCC |
| *fabp2* | GTCATAGAGGGAGACACGCT | CTATAGCTCTGTACAAGTTCACCATTA |
| her4 | ACTGAACACAAGACACACAGC | CATGGGCTTTCTCAGCTTGTT |
| her6 | AAAACGGCTTCGGAACACAG | AGTTTAGAGTGTCTGGAGCTATCTT |
| hey2 | CCAAGTTGGAGAAAGCGGAAAT | GAGAATGAGCGTCGAAATATCCTTT |
| *axin2* | GGGTCTGTCTGCCAAAACAC | CTGCGTCTGTAGGACCTGTA |
| *tcf7* | CACGATGGAGGGATGTATAA | CTTGTTGGAAGATGGAGAGA |
| *lef1* | CCGCACAAGGAGCAAATCTT | TAGCTGCGTCGTGACTGTTA |
| *prkaa1* | GCACATGAATGCCAAGATTGC | CCCTGCGTATAACCTTCCAGA |
| *ptch1* | GCGGCGGATTTGGACTATTT | CAGTCGCATTCCCCTCTGAT |
| *gli1* | ACACACTCCAATGAGAAACCG | GGTCCGTGTACCGTCTTTAC |
| *atp6v1e1a* | TTCGCATCGACGAGAACACT | GATGTCAGGCATCATCTGTTGT |
