# Supplementary material for: Loss of zebrafish atp6v1e1b, encoding a subunit of vacuolar ATPase, recapitulates human ARCL type 2C syndrome and identifies multiple pathobiological signatures
Source: PLoS Genet. 2021 Jun 18;17(6):e1009603. doi: 10.1371/journal.pgen.1009603 (PMC8244898; doi:10.1371/journal.pgen.1009603)
Supplement: S1 Text — (DOCX) [file pgen.1009603.s015.docx]

**Supporting information**

**Assay of motor function, survival, and lysosomal staining.**

Touch-evoked escape response was measured at 3 dpf using a scale from 1 to 4: 1, no movement; 2, local muscle contraction of the embryo; 3, short distributed swim movement; 4, normal swim movement towards the edge of the Petri dish. Zebrafish were touched with the tip of a P10 pipette at the end of their tail. Zebrafish mortality was scored based on cardiac arrest and tissue degradation. Survival analysis was plotted using Kaplan-Meier curves. For lysosomal imaging, zebrafish larvae at 3 dpf were incubated with LysoTracker Red DND-99 (Life Technologies, Carlsbad, CA, USA) which contains fluorescent acidotropic probes for labeling and tracking of acidic organelles. Zebrafish larvae were subsequently washed and were embedded in 0.8% seaPlaque low melting agarose (Lonza, Basel, Switzerland) supplemented with 160 mg/L tricaine (Sigma-Aldrich, Saint Louis, MI, USA). Brain area was imaged using a Leica TCS LSI zoom confocal microscope (Leica Microsystems, GmbH, Wetzlar, Germany). Image reconstruction was performed with Fiji software [1].

**Immunofluorescence**

Cryosections were prepared as previously described [2]. Briefly, larvae of 4 dpf were fixed in 4% PFA in PBS overnight (ON). Fixed larvae were de- and rehydrated in serial MeOH-TBST dilutions. Rehydrated larvae were subsequently stored in 30% sucrose for 1h, Next, the samples were incubated in 15% fish gelatin/25% sucrose ON. Larvae were embedded in Tissue-Tek O.C.T. (Optimum Cutting Temperature) compound (Sakura Finetek Europe, Alphen aan den Rijn, The Netherlands), frozen on dry ice, and cut into longitudinal 12 µm cryosections. Sections were dried on RT for 1h and postfixed in 4% PFA for 20 minutes. To reduce nonspecific binding of antibodies, slides were incubated with blocking solution (10% normal goat serum [Thermo Fisher Scientific, Waltham, Massachusetts, USA] and 0.1% Triton X-100 in PBS) at RT in a humidified atmosphere. Slides were washed 6 times in PBS and subsequently incubated with the primary antibodies (anti-Rab5 antibody (1:100, C8B1, Cell Signaling Technology (CST), Danvers, Massachusetts, USA), monoclonal rabbit anti-Rab7 antibody (1:100, D95F2, CST), and polyclonal rabbit anti-LAMP1 (1:1000, ab24170, Abcam, Cambridge, United Kingdom) at 4°C ON. Antibodies were diluted in blocking buffer. Slides were washed 6 times in PBS followed by an 2h incubation with the fluorescent secondary antibody diluted in blocking buffer (Goat Anti-Rabbit IgG, DyLight^TM^ 633, 1:200, Life Technologies). Slides were mounted on Vectashield with DAPI in order to visualize nuclear staining. Datasets were collected on an observer Z.1 microscope equipped with a yokogawa disk CSU-X1 (Zeiss, Zaventem, Belgium). Images were captured using a 63x Pln Apo/1.4 oil objective in combination with a Photometrics sCMOS Prime 95B (Teledyne Photometrics, USA) camera. Per condition z-stacks were created with a z-interval of 0.175 µm. Parameters such as detector gain, laser intensity, exposure time, and image post-processing were kept consistent between the different conditions. Image reconstruction was performed with ZEN Blue and Fiji software [1].

**Whole-mount in situ hybridization**

cDNA from sense and antisense gBlocks were amplified by PCR, followed by subsequent *in vitro* transcription targeting. High-resolution whole-mount in situ hybridization (WISH) of zebrafish embryos was carried out as previously described [3]. *Atp6v1e1a* and *atp6v1e1b* antisense probes were used to study expression at 24, 48, 72, 120, and 168 hours post fertilization (hpf). Stained specimens were analyzed with a Leica M165 FC Fluorescent Stereo Microscope (Leica Microsystems, GmbH, Wetzlar, Germany).

**Whole Mount Staining with Alizarin Red S**

Alizarin Red staining of 12-month-old adult zebrafish and respective WT controls was performed as previously described [4]. Stained specimens were analyzed for ectopic bone with a Leica M165 FC Fluorescent Stereo Microscope (Leica Microsystems).

**Drug administration**

Compounds were administered to zebrafish embryos at 1 dpf, after chorion removal in E3 embryo medium (containing 5 mM NaCl, 0.17 mM KCl, 0.33 mM CaCl_2_, 0.33 mM MgSO_4_, and 50 mM HEPES pH 7.1) in a Petri dish. In cases in which drugs were dissolved in DMSO dilutions were made so the final DMSO concentration did not exceed 1% in the Petri dish with the exception of nicotinic acid. Nicotinic acid was dissolved in NaOH. The final NaOH concentration did not exceed 0.03% in the Petri dish. The relevant vehicle control was used for each experiment. Drugs are listed in **S4 Table**.

**Supporting references**

1. Schindelin J, Arganda-Carreras I, Frise E, Kaynig V, Longair M, Pietzsch T, et al. Fiji: an open-source platform for biological-image analysis. Nature methods. 2012;9(7):676-82. Epub 2012/06/30. doi: 10.1038/nmeth.2019. PubMed PMID: 22743772; PubMed Central PMCID: PMCPMC3855844.

2. Ferguson JL, Shive HR. Sequential Immunofluorescence and Immunohistochemistry on Cryosectioned Zebrafish Embryos. Journal of visualized experiments : JoVE. 2019;(147). Epub 2019/06/04. doi: 10.3791/59344. PubMed PMID: 31157768; PubMed Central PMCID: PMCPMC7291005.

3. Thisse C, Thisse B. High-resolution in situ hybridization to whole-mount zebrafish embryos. Nature protocols. 2008;3(1):59-69. Epub 2008/01/15. doi: 10.1038/nprot.2007.514. PubMed PMID: 18193022.

4. Sakata-Haga H, Uchishiba M, Shimada H, Tsukada T, Mitani M, Arikawa T, et al. A rapid and nondestructive protocol for whole-mount bone staining of small fish and Xenopus. Scientific reports. 2018;8(1):7453. Epub 2018/05/12. doi: 10.1038/s41598-018-25836-4. PubMed PMID: 29748567; PubMed Central PMCID: PMCPMC5945591.
