# Supplementary material for: Loss of zebrafish atp6v1e1b, encoding a subunit of vacuolar ATPase, recapitulates human ARCL type 2C syndrome and identifies multiple pathobiological signatures
Source: PLoS Genet. 2021 Jun 18;17(6):e1009603. doi: 10.1371/journal.pgen.1009603 (PMC8244898; doi:10.1371/journal.pgen.1009603)
Supplement: Attachment — Submitted filename: Response to the reviewers.docx [file pgen.1009603.s016.docx]

**Reviewer #1**

Please find enclosed the comments to the research article “Loss of a subunit of vacuolar ATPase identifies unexpected biological signatures of reduced organelle acidification in vivo, PGENETICS-D-20-01563” submitted for publication in PLOS Genetics by Bert Callewaert and colleagues. The paper comprises a set of interesting data important for a better understanding of cutis laxa conditions from a pathophysiological perspective. Furthermore, these date show that interfering with v-ATPase function in vivo also changes several pathways not obviously connected to this protein complex.

Major points:
The title to me would benefit from mentioning the organism analyzed and the deficient gene.

We thank the reviewer for the suggestion. We changed our title to ‘Loss of zebrafish *atp6v1e1b*, encoding a subunit of vacuolar ATPase, recapitulates human ARCL type 2 syndrome and identifies multiple pathobiological signatures of reduced vesicular acidification’.

Introduction.
This section provides the reader with the function of the v-ATPase in general, with a broad list of mendelian conditions due to pathogenic variants affecting v-ATPase subunits and many pathways connected to this protein complex function. Some of these information’s are not necessary for the present paper and should be removed. Additionally, some of the knowledge presented here, is not further used throughout the manuscript/discussion and some of the pathways mentioned and very likely related to the multisystem phenotype in cutis laxa (e.g. AMPK) were unfortunately not investigated in the present in vivo model. Additionally, no information on tissue specific expression/assembly of different v-ATPase components have been provided which could be important for the discussion of the results. I recommend a restructuration of the introduction to allow the reader a better understanding of the project performed and to provide a more focused basis for the data and their discussion.

We agree with the reviewer that the manuscript benefits from a more logical structure of the introduction. Since we have included more information regarding the non-canonical pathways that are influenced by v-ATPAses, we decided to retain this part of the introduction. In the revised manuscript, we investigated the mTOR signaling pathway on protein level and analyzed gene expression for AMPK, Wnt and Notch responsive genes by RT-qPCR. We excluded the broad list of Mendelian disorders and the cancer link with v-ATPases as this information is superfluous.

It is known that *ATP6V1E1*, *ATP6V1A* and *ATP6V0A2* are ubiquitously expressed (heart, esophagus, colon, cervix, brain, bladder, adipose, skeletal muscle, prostate, placenta, ovary, lung, liver, kidney, trachea, thyroid, testes, spleen, small intestine, fibroblasts, skin, and tendon) while the expression of *ATP6V1E2* is restricted to testicular tissue. However, we did not specify this information in the introduction of the revised manuscript since we want to focus on the tissue specific expression of the zebrafish orthologs in the results section (S1 Fig.) and discussion.

Results
- In supplementary figure 1, the effect of the mRNA expression in both zebrafish lines have been analyzed. Additionally, both lines lead to a strong reduction of the mRNA. Have the authors tested the protein expression? I know about the problem that sometimes no working antibodies are available for zebrafish, however, ATP6V1E1 is highly conserved. Maybe one of the described antibodies work.

To address the reviewer’s comment, we tried to optimize 3 different antibodies:

- Anti-ATP6V1E1 antibody [EPR19602] (ab201468) Rabbit mAb
- Anti-ATP6V1E1 antibody (ab111733) Rabbit pAb
- Anti-ATP6V1E1 antibody (ab102609) Rabbit pAb

Only anti-ATP6V1E1 antibody (ab111733) Rabbit pAb showed sufficient specificity and provided reliable results in zebrafish. We observed a reduction in atp6v1e1 protein expression levels in *atp6v1e1b*-deficient zebrafish compared to control siblings, which was more pronounced in *atp6v1e1b*^cmg78/cmg78^ compared to *atp6v1e1b*^hi577aTg/hi577aTg^ zebrafish. Previous studies claimed that the large proviral insertion yielded an abnormal N-terminal transcript fragment that most likely would not be translated into protein, which our current data partly refutes. We added these data in the results (S2 Fig.) and discussed these observations in relationship with the observed phenotypes in the revised manuscript.

- Have the authors observed a less severe phenotype in the line with the insertion within the 5’UTR. For example, the vascular phenotype seems to be less severe. There is still some mRNA left which, due to the fact that the ORF should be intact, might get translated into active protein. Can the authors comment on that and include some words into the discussion?

Based on our data, we cannot conclude that *atp6v1e1b*^hi577aTg/hi577aTg^ has a less severe phenotype compared to *atp6v1e1b^cmg78/^*^cmg78^. The phenotype is almost identical apart from several small exceptions. The exceptions are most likely due to different residual protein expression of atp6v1e1. We confirmed that more residual protein is present in *atp6v1e1b*^hi577aTg/hi577aTg^ compared to *atp6v1e1b^cmg78/^*^cmg78^, as correctly hypothesized by the reviewer. In the discussion of the revised manuscript we have further elaborated on the major phenotypic differences between both models (complex N-glycan abnormalities, Golgi morphology, and mitochondrial response to Bafilomycin A1), in light of the different protein levels of atp6v1e1 measured in both lines.

- A more intuitive presentation of the EM data showing the matrix alterations would be of help. Can the authors show all the pictures in the same orientation?

The revised manuscript shows EM data highlighting matrix abnormalities in the same orientation. This will make the matrix abnormalities more obvious to the reader. Moreover, we indicated the basement membrane in dotted lines to emphasize the bent basement membrane structures in *atp6v1e1b*^cmg78/cmg78^.

- Complex glycan structures: It is not clear why some of the lines investigated (3/4) do not show any presence of these structures. Is this only due to the scaling of the y axis or are these structures not present at all. If so, why is the glycosylation phenotype more pronounce in the line carrying the frameshift (see above, differences of the manifestations) Can the authors discuss this point also with respect to potential Golgi abnormalities (see below)?

We thank the reviewer for this comment and acknowledge that the description of these results was not entirely clear. Some complex N-glycan structures are not present in control siblings and *atp6v1e1b*^hi577aTg/hi577aTg^ zebrafish. We now amended the results with further data. Our EM data (see below) do show dilated Golgi in *atp6v1e1b^cmg78/^*^cmg78^ while the few remaining Golgi appear normal in *atp6v1e1b*^hi577aTg/hi577aTg^ (Fig.2). It is tempting to speculate that the sequential steps in N-linked glycosylation are disrupted in dilated Golgi which could cause competition among enzymatic modifications and generate a heterogenous glycan mixture as observed in the *atp6v1e1b^cmg78/cmg78^* line. The exact mechanisms underlying the milder glycosylation abnormalities in the *atp6v1e1b*^hi577aTg/hi577aTg^ line, associated with a less disrupted Golgi phenotype, remain to be elucidated in future studies, but likely involve some residual activity from the remaining atp6v1e1b protein expressed in these zebrafish.

- The point about the endosomal and lysosomal alterations is to strong and not conclusively supported by the data. The alteration on EEA1 is impressive, however, the changes on Rab5 and Lamp1 seem to be minor. Additionally, have the author investigated the localization/morphology of these vesicles.

We have softened our conclusions regarding the effect of *atp6v1e1b*-deficiency on the maturation of the endosomal pathway since our data are not unequivocal. New confocal microscopy data obtained from cryosections of *atp6v1e1b*^hi577aTg/hi577aTg^, *atp6v1e1b^cmg78/cmg78^* and WT controls (S4 Fig.) did however demonstrate alterations in localization and structure of these vesicles. We included these extra data in the revised manuscript.

-Immunostaining of these markers (especially EEA1 and Lamp1) should be possible. The lysotracker staining is impressive. However, it is not immediately clear which part of the larvae was imaged. Could the authors show the structure by dotted lines within the picture or is a quantification possible? I do agree about alteration within the endosomal and lysosomal system, which is somewhat expected, however, I suggest weakening the point a bit.

In the revised manuscript, we have included additional immunostaining data, and we have added a schematic presentation of the area scanned by confocal microscopy (S3 Fig.). Moreover, we quantified the number of acidified vesicles (endosomes, lysosomes, exosomes, ...) detected by the lysotracker stain as suggested by the reviewer. As noted above, we have weakened our conclusions regarding the maturation of the endo(lyso)somal pathway, although we do have extra immunolocalization evidence of differences between early and late endosomal vesicles in *atp6v1e1b*^hi577aTg/hi577aTg^, *atp6v1e1b^cmg78/cmg78^* and WT controls.

- The analysis of the HIF target genes and the rescue experiments are interesting. I understand the point that the analysis of the iron “uptake” is crucial. However, from my point of view this seems not be altered at all. This to me leads to some lack of clarity within the complete manuscript. Focusing on the strongly altered pathways identified would lead to more clarity.

The HIF1a-pathway is indeed not altered, as rightfully remarked by the reviewer. Nevertheless, we felt that this is an important observation in light of the published *in vitro* data which shows that inhibition of v-ATPases induced an upregulation of HIF-target genes. However, we shifted our focus in the revised manuscript more towards the analysis of the non-canonical pathways modulated by v-ATPases. Therefore, we moved the HIF1a data and the *in vitro – in vivo* discrepancy to the supplemental figures (S5 Fig.).

- The transcriptome analysis provides a lot of relevant information into the mechanisms leading to CL. The authors state, that the main candidate genes mentioned in the introduction were only minorly changed. Have the authors validated some of the candidates (OXPHOS….) and these initially mentioned genes pointing into distinct pathways (mTOR, AMPK) by qPCR? Additionally, changes in these pathways are not necessarily detectable via transcriptomics. For Notch, mTOR and AMPK signaling, good antibody kits are available which could help to identify potential misregulations/activations of them. Did the authors perform comparative proteomics?

We agree with the reviewer that changes in mTORC1 and AMPK activity are not necessarily detectable via transcriptomics. We now include information regarding protein expression and phosphorylation of p70 S6K, p85 S6K, and p31 S6K in the revised manuscript (Fig.3). We observed evidence of an increase in mTORC1 activity in *atp6v1e1b*-deficient zebrafish, which is discussed in the revised manuscript.

We have tried to optimize different antibodies from Cell Signaling Technology to address AMPK activity on protein level (phospho-AMPK/AMPK ratio). Unfortunately, these antibodies induced multiple aspecific bands on the PVDF membrane hampering interpretation of the results.

- Anti-Phospho-AMPKα (Thr172) (40H9) Rabbit mAb (#2535)
- Anti-AMPKα (F6) Mouse mAb (#2793)
- Anti–AMPKα (Thr-172) Rabbit mAb (# 2535)

To further address the reviewer’s comment, we have added new qPCR data demonstrating the gene expression levels of Wnt-responsive, Notch-responsive and AMPKα-responsive genes in the revised manuscript. We did not perform comparative proteomics at this point in our study, taking into account the complexity of this type of experiment in zebrafish. Nevertheless, the reviewer raises an excellent point and we will implement proteomic profiling in future experiments regarding KO ZF models of v-ATPase subunits.

- The lipidomics data are interesting. As a non-expert in zebrafish biology I was wondering where developmentally the origin of the yolk is. Due to the fertilization process this is very likely produced within the fish egg. If so, are these finding rather due to altered egg synthesis/composition of the yolk provided by the heterozygous mother? Have the authors found similar changes in specific cell types of the larvae itself?

The reviewer raises a very interesting point. Zebrafish rely on yolk contents, which indeed are provided by mother during oogenesis, for nutrition throughout development until 5 days post fertilization, when the active feeding behavior of the zebrafish larvae starts. Because of this reliance on the yolk, zebrafish embryos up to 5 dpf can be considered a ‘‘closed system,’’ meaning that their nutrient intake is uninfluenced by outside factors. It is known from literature that lipids are processed within the yolk prior to mobilization to the embryonic body during early embryogenesis. The levels of the different lipid classes also change during embryonic development. Unfortunately, we cannot exclude potential contributions of maternal lipids to the interpretation of the results from the lipidome analysis. Since the *atp6v1e1b*-deficient zebrafish are not viable, maternal zygotic knockout embryos cannot be generated. However, any differences identified in our experiment are related to the *atp6v1e1b*-deficiency as the measurements were obtained at equal developmental time points and between mutant zebrafish and WT siblings. We did not perform lipidome analysis on specific cell types of the *atp6v1e1b*-deficient zebrafish as we decided to investigate the intact embryos to minimize the amount of potentially artefact-inducing disturbances. It is known in literature that deyolking zebrafish, and therefore removing most maternally-derived mRNA and protein contributions prior to sample collection can cause disruption of other biological processes by itself. We took notice of this issue in the discussion.

Rahlouni F, Szarka S, Shulaev V, Prokai L. A Survey of the Impact of Deyolking on Biological Processes Covered by Shotgun Proteomic Analyses of Zebrafish Embryos. Zebrafish. 2015;12(6):398-407. doi:10.1089/zeb.2015.1121

- The OCR data presented are interesting. To me, and in comparison, to some literature the reaction to Rotenone/Antimycin seems relatively slow. Can the authors state on this?

Our experiences in using Seahorse XF24 for zebrafish purposes have taught us that the Rotenone/Antimycin A reaction is slower than in cell culture systems. Moreover, it can be difficult to compare OCR responses between different experiments as these can deviate significantly between different conditions (number of zebrafish per well, developmental timepoint). We optimized OCR response curves with different concentrations of rotenone and antimycin at different developmental conditions. The response time to rotenone/antimycin can be influenced by a delayed uptake, potentially due to prior exposure to other mitochondrial poisons (oligomycin or FCCP) which might impact the general condition of the zebrafish embryos. This is also likely the cause why the data can become less reliable toward the end of the experiment, as illustrated by the gradual increase in OCR observed in some cases. Therefore, we have decided to exclude the final 40 min of the measurement (after 30 min exposure to rotenone/antimycin). We have therefore updated Fig. 6, showing responses up to 260 min.

- One cellular feature observed in ATP6V1E1 related CL was a dilated Golgi. Did the authors found any evidence for this in the EM data or using immunolabeling?

We reanalyzed the EM data of WT and *atp6v1e1b*-deficient zebrafish based on the suggestion of the reviewer. Indeed, the Golgi apparatus was more frequently visualized in WT zebrafish compared to *atp6v1e1b*-deficient zebrafish. Moreover, the Golgi appeared clearly dilated in *atp6v1e1b^cmg78/cmg78^* zebrafish, while they had a normal appearance in *atp6v1e1b^hi577aTg/hi577aTg^* zebrafish. We added these new data to the results section (Fig. 2) and discussion in the revised manuscript.

Discussion:
The discussion at many points recapitulates the findings presented within the results section. Additionally, the strong focus on the expected, but not found iron related phenotypes gives the paper a strong imbalance towards this point. Other features observed (e.g. hypotonia) were not discussed at all especial in relation to the mitochondrial alterations. The discussion about the relation of v-ATPase and cancer is interesting, however, to me it is not clear why this point is so extensively discussed.

We thank the reviewer for this feedback. We took extra care to rewrite the discussion more in line with the major findings of our results and changed the focus to the altered pathways. As a considerable amount of extra data is now included in the manuscript, we decided to exclude the discussion regarding the relation of V-ATPases and cancer as this no longer fits the scope of the revised paper.

Finally, the data presented are interesting and relevant. I recommend to restructure the complete manuscript with a focus on the altered pathways identified. I suggest starting with the presentation of the phenotype, glycosylation abnormalities and continue right away with the transcriptomics. From there, the argumentation towards the mitochondrial alterations, for example, are clear. All the findings should be discussed in more detail in relation and in comparison, to physiological processes and to the human disease.

We thank the reviewer for the valuable comments and suggestions and hope that we have addressed this point satisfactorily in the thoroughly revised manuscript.

Minor points:
Sometimes it is not immediately clear how many animals have been subjected to each analysis and how many technical replicates have been performed. This should be carefully checked throughout all datasets

We have taken extra care in listing the number of technical replicates in each figure legend of the revised manuscript. We have also ensured that the number of zebrafish larvae included in the experiments, which often contains pooled zebrafish larvae, are properly described in the material and methods section.

In some datasets two WT lines have been analyzed whereas the second line is missing in some other sets of data. Why haven’t both control lines been used consequently?

For the majority of the experiments we used mutant zebrafish and their respective WT siblings, hence the use of two separate WT controls. We deviated from this approach for our transcriptome analysis in order to reduce the number of occupied lanes on the HiSeq NGS sequencer (Illumina). We believe that this approach can be justified since the respective WT siblings from the two mutant zebrafish lines have a very similar genetic background. The proviral insertion line which was obtained from ZIRC was backcrossed for multiple generations (and obtained 87.5% AB background) to the AB zebrafish line in our zebrafish facility, which was used to generate the CRISPR mutant. Therefore, we do not expect major differences between the WT zebrafish lines. In line with the transcriptomic experiments, subsequent qPCR experiments also included the same WT zebrafish control.

In some cases, the figure reference within the text doesn’t fit to the data presented in the figures (Example: In addition, downregulation of elnb expression (Figure 2A+2B+) occurred in both mutant zebrafish lines)

We thank the reviewer for notifying us of this mistake. In the revised manuscript, we have changed the figure legend to refer to the appropriate subpanel.

**Reviewer #2**

The authors describe their findings on two Atp6v1eb zebrafish models, one that they generated using CRIPR-Cas9, and one obtained from ZIRC. Both mutant lines show severe phenotype that include reduced survival rates, impaired touch-evoked escape response, maxillary and mandibular hypoplasia, vascular abnormalities, and some indication of cardiac abnormalities. Overall the phenotypic characterization of the two genetic mutants are useful for future therapeutic explorations. The ~omics profiling is excellent.

Major Comments:
1. The authors have shown increased total EEA1 and ~reduced Rab7 levels by western blot. These results, however, do not necessarily suggest impaired endosomal maturation, and could not support a strong interpretation that deficiency of v-ATPase plays a prominent role in the maturation of endo(lyso)somes, nor that they have shown mechanistically that in vivo genetic depletion of atp6v1e1b leads to .. reduced maturation of endosomal and lysosomal vesicles. They also refer to a paper that reviews vesicular acidification and v-ATPase, among other things, but no association with endolysosomal maturation. To support the conclusion of reduced endolysosomal maturation, the authors would need to show the levels membrane-bound endosomal markers as compared to cytosolic membrane markers. An alternative would be to show confocal images of these markers, or to show accumulation of cargos that go through the endosomal pathway.

Thank you for raising this important point. In order to provide direct evidence for impaired endolysosomal maturation impaired using the currently available zebrafish models, we performed confocal imaging of Rab5, Rab7, and LAMP1 markers on cryosections of *atp6v1e1b*^hi577aTg/hi577aTg^, *atp6v1e1b^cmg78/cmg78^* and WT controls (S4 Fig.). Our data show clear changes in tissue specific expression and localization of these markers, supporting alterations of the endo(lyso)somal pathway. This, in addition to the striking imbalance in Rab5 to Rab7 ratios indicates abnormal vesicle maturation. Nevertheless, we realize that the current evidence does not allow us to draw a definitive conclusion on the exact impact of *atp6v1e1b* deficiency on endosomal vesicle maturation. In the revised manuscript we therefore softened this point in the discussion. We plan to pursue the analysis of membrane-bound and cytosolic endosomal markers in *atp6v1e1b*-deficient zebrafish in future research, using hypomorphic zebrafish models with increased survival as this will allow us to obtain sufficient tissue-specific material for analysis and to minimize potential secondary effects.

2. Although a hypothesis that LOF of Atp6v1eb in zebrafish leads to reduced acidification is not unexpected, the authors only showed lysotracker staining. This result will be strengthened by measurement of pH in lysosomes, or endolysosomal compartments.

We acknowledge the reviewer’s concern, but unfortunately measuring pH in zebrafish is not straightforward. Based on literature, we have not found a fast and easy way to perform these experiments, with the exception of using the LysoTracker Red staining which we show here. We also tried to optimize LysoSensor™ Yellow/Blue DND-160, a ratiometric probe that can be used in addition to LysoTracker Red to measure the pH of acidic organelles. The advantage of the LysoSensor dye compared to LysoTracker Red is that this dye produces blue fluorescence in neutral environments, but changes to yellow fluorescence in more acidic environment, enabling monitoring of vesicles. Unfortunately, we were not able to use this staining in our zebrafish models as it did not penetrate the vesicles.

For follow-up projects we want to use a fluorescent-based reporter system using the genetically encoded pH-sensitive GFP variant pHluorin which is one of the most convenient and easiest ways to measure intracellular pH. It has been used to study cytosolic pH as well as organellar pH, including mitochondria and peroxisomes. Very recently, a new pH biosensor has been designed specifically for lysosomal measurements, based on a fusion with LAMP1 (pH Lysosomal Activity Reporter, pHLARE) We are aiming to create transgenic zebrafish lines ubiquitously expressing the pHluorin and pHLARE reporters in the *atp6v1e1b* mutant background in order to assess the impact on cytosolic and lysosomal pH. The generation and validation of these new transgenic lines will be pursued in our follow-up studies.

3. The authors showed that FAC treatment did not rescue the survival rates of the two zebrafish mutants; also their data seem to indicate that there is no induction of HIF. It isn’t clear to me which dataset the authors used to support their initial hypothesis in pursuing this pathway, other than the previous report on ATP6V0 subunit. Are the authors thinking that the entire V-ATPase complex is destabilized upon loss of Atp6v1eb in zebrafish, or do they have results to show this? Do they have data showing reduced iron levels in their zebrafish models?

Thank you for raising this issue as we now realize that this point needs further clarification. Besides the canonical (acidification of vesicles) and non-canonical (modulation of mTOR, Wnt, Notch, and AMPK activity) functions, we found that multiple studies described a HIF1a-mediated response upon genetic and pharmacological inhibition of v-ATPases *in vitro*. We refer to these studies (see below) which are highlighted in the introduction.

Yambire KF, Rostosky C, Watanabe T, Pacheu-Grau D, Torres-Odio S, Sanchez-Guerrero A, et al. Impaired lysosomal acidification triggers iron deficiency and inflammation in vivo. eLife. 2019;8. Epub 2019/12/04. doi: 10.7554/eLife.51031.

Miles AL, Burr SP, Grice GL, Nathan JA. The vacuolar-ATPase complex and assembly factors, TMEM199 and CCDC115, control HIF1alpha prolyl hydroxylation by regulating cellular iron levels. eLife. 2017;6. Epub 2017/03/16. doi: 10.7554/eLife.22693.

Weber RA, Yen FS, Nicholson SPV, Alwaseem H, Bayraktar EC, Alam M, et al. Maintaining Iron Homeostasis Is the Key Role of Lysosomal Acidity for Cell Proliferation. Molecular cell. 2020;77(3):645-55 e7.

In the revised manuscript, we have shifted our focus away from the HIF1a-mediated response as we did not observe any alterations in this pathway *in vivo* in the *atp6v1e1b*-deficient zebrafish (see also the comments of reviewer 1). Nevertheless, we still would like to highlight the discrepancy in HIF1a results obtained in the published *in vitro* systems vs our *in vivo* models in the revised manuscript, which is why we are showing our data regarding iron homeostasis in the supplement (S5 Fig.). We did not directly measure iron levels in the zebrafish larvae, but we have used o-dianisidine staining of hemoglobin levels. This is a staining routinely used in anemia research to assess hemoglobinization, which is heavily dependent on iron homeostasis. Based on this staining we concluded that there is no difference in general iron levels in the mutant zebrafish embryos.

We do have evidence that loss of *atp6v1e1b* destabilizes the entire v-ATPase complex. Van Damme T *et al.* performed complexome profiling on dermal fibroblasts of patients harboring pathogenic missense variants in *ATP6V1E1* and *ATP6V1A* and controls. Fully assembled v-ATPase and the separate V1 and V0 domains were detected in controls. In patient’s dermal fibroblast the overall abundance of the V1 subunits and the fully assembled V-ATPase complex was markedly reduced, but the amount of V0 domain separately was either increased or hardly changed. These *in vitro* results indicate that loss of the peripheral stalk, the E subunit, leads to a loss of the V1V0 domain assembly.

4. As a follow up to the previous question, I do not follow the seemingly surprising finding that there is a lack of hypoxia-mediated response in vivo. In human patients, there is an increased in trisialotransferrin, but I do not see similar data in the zebrafish – is transferrin abnormalities also seen in mutant Atp6v1eb zebrafish? In addition, there is no data showing detection of intracellular iron amounts in zebrafish mutants, that could potentially lead to HIF-mediated response.

We show in our *atp6v1e1b*-deficient zebrafish model that there is no defect in the hypoxia-mediated response (S5 Fig.). This observation is in contrast to what has been previously reported by multiple studies *in vitro*, which also showed that this response could be reverted by supplementation of iron*.* To verify whether iron supplementation would have a beneficial effect *in vivo*, we added FAC to our *atp6v1e1b*-deficient zebrafish, but without success (S5 Fig.). We did observe that several hemoglobin genes (*hbbe1.3*, *hbae1.1*, and *hbbe1.1*) were regulated differently although hemoglobin levels were similar between *atp6v1e1b*-deficient zebrafish and WT controls.

Similar to mutations in *ATP6V0A2*, mutation in *ATP6V1E1* and *ATP6V1A* might lead to N-glycosylation abnormalities. As the reviewer correctly noticed, routine glycosylation screening by means of transferrin isoelectric focusing was performed in the paper of Van Damme T *et al.* However, this screening was unrelated to possible hypoxia-mediated responses. The available *in vitro* data rather points to a direct effect of decreased vesicle acidification on iron uptake. We do however recapitulate the changes in relative levels of different N-glycans in our *atp6v1e1b*-deficient zebrafish model via N-glycome analysis (Fig. 2), but did not focus specifically on transferrin sialylation.

5. The authors wrote, “Our data suggest alternative routes, bypassing the transferrin receptor pathway, to deliver iron to mitochondria in vivo” – but there is no evidence to suggest this.

We agree that at this point we have no evidence to suggest this hypothesis. We have therefore excluded this sentence from the conclusions in the revised manuscript, and plan to follow up on this possibility in future studies.

Minor comments:
1. Mitochondrial profiling
a. Upon treatment of oligomycin, the OCR levels in the mutants were increased. How do the authors explain these results, given the reduced baseline OCR levels (which could indicate low ATP production?

We elaborated on this observation in the discussion of the revised manuscript. A reverse direction or operation of the mitochondrial ATP synthase (F-ATPase) has been previously observed when mitochondrial dysfunction is induced by disrupting electrochemical membrane potential. This mechanism could explain the unexpected OCR increase in *atp6v1e1b*-deficient zebrafish following administration of oligomycin. Indeed, given the evolutionary relationship between v-ATPase and F-ATPase, alteration of the E1 subunit of v-ATPase may directly impact F-ATPase function. A similar effect has recently been observed in trypanosomes, where v-ATPase depletion was shown to affect F-ATPase coupling. However, the functional interrelationship between v-ATPase and F-ATPase is beyond the scope of this work and will be investigated in detail in future studies.

Baker N, Hamilton G, Wilkes JM, Hutchinson S, Barrett MP, Horn D. Vacuolar ATPase depletion affects mitochondrial ATPase function, kinetoplast dependency, and drug sensitivity in trypanosomes. Proceedings of the National Academy of Sciences of the United States of America. 2015;112(29):9112-7. Epub 2015/07/08. doi: 10.1073/pnas.1505411112. PubMed PMID: 26150481; PubMed Central PMCID: PMCPMC4517229.

2. Glycosylation profile:
a. What’s the significance of high mannose species in the mutant zebrafish lines? Does this correlate to an abnormal Golgi function since these species are glycoforms that indicate pre-Golgi processing?

The increase in 9-mannose N-glycans indeed correlates with an abnormal Golgi function. N-linked glycosylation is initiated in the endoplasmic reticulum, with subsequent glycan processing during transport through the Golgi. Within the Golgi, an interplay of glycosidase-mediated mannose trimming and monosaccharide additions via glycosyltransferases generates N-glycans classed as oligo-mannose (five to nine mannoses), hybrid, and complex structures. Since disruption of the v-ATPase leads to reduced acidification, it is tempting to speculate that the enzymatic function of mannosidases in the *cis*-Golgi compartment will be reduced and therefore alters subsequent trimming towards 5-mannose N-glycans. In the revised manuscript, we elaborated on this issue in the discussion on the glycosylation profile of *atp6v1e1b*-deficienct zebrafish.

Fisher P, Spencer H, Thomas-Oates J, Wood AJ, Ungar D. Modeling Glycan Processing Reveals Golgi-Enzyme Homeostasis upon Trafficking Defects and Cellular Differentiation. Cell reports. 2019;27(4):1231-43 e6. Epub 2019/04/25. doi: 10.1016/j.celrep.2019.03.107. PubMed PMID: 31018136; PubMed Central PMCID: PMCPMC6486481.

Stanley P. Golgi glycosylation. Cold Spring Harbor perspectives in biology. 2011;3(4). Epub 2011/03/29. doi: 10.1101/cshperspect.a005199. PubMed PMID: 21441588; PubMed Central PMCID: PMCPMC3062213.

b. The data seem to indicate that changes in complex glycans are more than the change in high mannose species – is this the case?

We would like to draw the reviewer’s attention to the different scales of the Y-axes in the two graphs comparing oligo-mannose N-glycans with complex N-glycans. The absolute change in 9-mannose glycans between *atp6v1e1b*-deficienct zebrafish and WT is approximately an order of magnitude higher than the change in complex N-glycans between *atp6v1e1b*-deficienct zebrafish and WT. Nevertheless, relatively speaking the *atp6v1e1b^cmg78/cmg78^* embryos show a much stronger contribution of Neu5Gc than Neu5Ac terminal species compared to their control siblings, possibly related to a different regulation of cytidine monophospho-N-acetylneuraminic acid hydroxylase (CMAH).

c. How would the author explain the differences in glycosylation pattern between the two mutant zebrafish lines? And Why is there a difference in the terminal sialic acid in the two mutant lines

We reanalyzed our EM data, and found a reduced abundance of Golgi apparati in both *atp6v1e1*-deficient zebrafish lines (Fig. 2). In addition, the residual Golgi still detected in *atp6v1e1b^cmg78/^*^cmg78^ zebrafish showed a dilated phenotype, while the remaining Golgi in *atp6v1e1b*^hi577aTg/hi577aTg^ appeared normal. It is tempting to speculate that these dilated Golgi point to a stronger disruption of sequential enzymatic N-linked glycosylation modifications in *atp6v1e1b^cmg78/cmg78^*. Because the few remaining Golgi in *atp6v1e1b*^hi577aTg/hi577aTg^ appear to be intact, it is conceivable that this sequential N-linked glycosylation modification is not that severely altered in this zebrafish line. More specifically, it is likely that the terminal sialic acid conversion of Neu5Ac to Neu5Gc by CMAH is enhanced due to altered pH levels leading to predominant Neu5Gc as terminal sialic acid in *atp6v1e1b*^cmg78/cmg78^. The exact mechanisms underlying this difference between the two mutant lines remain to be elucidated in future studies, but they likely involve some residual activity from the remaining atp6v1e1b protein still expressed in the *atp6v1e1b*^hi577aTg/hi577aTg^ line.

Of note, while the difference in the complex glycan profiles between both mutant lines is interesting from a mechanistic perspective, the relevance for human biology is very limited since the CMAH gene has been inactivated during evolution just prior to human speciation. Nevertheless, we have expanded the discussion on this aspect of the glycosylation profile in the revised manuscript.

3. Lipidomics
a. How do the authors interpret the increased amounts of DCER, but not CER.

Ceramide synthesis occurs through the *de novo* synthesis pathway in the ER, after which it is translocated to the Golgi apparatus and further distributed to the plasma membrane and lysosomes. Since *atp6v1e1b*-deficient zebrafish show reduced and abnormal Golgi, translocation of ceramides might be reduced or delayed, leading to accumulation of CER and its direct precursor DCER. High levels of CER in the ER might induce a negative feedback loop on CER synthesis. Of note, we evaluated CER amounts in whole embryos. Hence, the overall amount of CER in the embryo is unaltered likely as a result of coupling between the *de novo* and salvage pathways and action of sphingomyelinase at the plasma membrane. Taken together, more experiments are clearly required to fully elucidate the mechanisms responsible for the lipid profile abnormalities observed in our zebrafish models, although this hypothesis is conceivable based on the known importance of acidic vesicles in these pathways. We have incorporated the above hypotheses into the revised discussion.

4. Staining/phenotyping
a. Some of the figures are difficult to appreciate. For example, I really do not see the dilated mitochondria. It is not clear if both Atp6v1eb mutants have similarly coiled basement membrane. It isn’t clear if the authors are referring to loss of collagen structures – because the images seem to show some disorganization of the bundles.

In the revised manuscript, we have adapted the phenotypic description of the dermis EM analysis (Fig. 2). We highlighted the basement membrane with dotted lines. The basement membrane is bent in *atp6v1e1b^cmg78/cmg78^* but not in *atp6v1e1b^hi577aTg/hi577aTg^*. We agree with the reviewer that we can only describe disorganized collagen bundles in the *atp6v1e1b*-deficienct zebrafish, and have adjusted the revised text accordingly. We also highlighted dilated mitochondria with dotted lines (Fig. 6).

b. The lysotracker staining isn’t clear. The authors would need to show what area of the fish is stained, and which cells are stained.

We have addressed this question by adding a schematic presentation of the area scanned by confocal microscopy. Moreover, we included a quantification of the acidified vesicles (endosomes, lysosomes, exosomes, ...) in S3 Fig.

c. Alcian blue showed maxillary and mandibular hypoplasia in both mutant zebrafish - Alcian blue shows reduced staining pattern – does this indicate bone cell abnormalities?

Indeed, in addition to the noted structural defects, *atp6v1e1b* mutants exhibited a reduction in the intensity of Alcian blue staining, suggesting changes in the expression of chondroitin sulfate proteoglycans. Endochondral ossification is not yet abundantly present at 5 days post fertilization, with only few bone cells formed at this developmental stage. In the revised manuscript, we added this observation to the results section.

d. Some of the data describing survival show datapoints up to 150 dpf. I thought the mutants only survived up to 8 dpf?

We thank the reviewer for noting this mistake. We meant 150 hpf and changed this in the revised manuscript.

5. How would you explain the reduced amount of collagen or elastin transcripts? Were these findings also seen in the transcriptomics studies?

Elastic fibers were almost completely absent in the reticular dermis of patients carrying a pathogenic missense mutation in ATP6V1E1. The few remaining elastic fibers were severely fragmented upon EM analysis. One hypothesis is that the elastin deposits aggregate due to altered pH in the Golgi apparatus and impair secretion and/or deposition in the ECM. We hypothesize that the initial secretion of tropoelastin still occurs but partly accumulates intracellularly and therefore, via a feedback mechanism, leads to compensatory lower gene expression. Altered collagen architecture might induce a feedback response enabling more protein expression to compensate the disruption in the collagen structure. Higher protein expression can then in turn decrease the collagen gene expression.

We could indeed corroborate these findings with our transcriptome dataset. We found a downregulation of *col11a1b*, *col16a1*, *col1a1a*, *col11a2*, *col12a1b*, *col1a2*, *col11a1a*, *col1a1b*, *col18a1a*, *col17a1a*, *col12a1a*, *col17a1b*, and *elnb* in *atp6v1e1b^cmg78/cmg78^* zebrafish larvae compared to WT. The transcriptional response of the collagen and elastin genes was less severe in *atp6v1e1b^hi577aTg/hi577aTg^* zebrafish. However, our transcriptome dataset showed a downregulation of *col5a2a*, *col9a1a*, *col6a1*, *col18a1b*, and *col28a2b* in this model. The qPCR results are obtained at 4 dpf in correspondence to the EM analysis, while the transcriptome study was performed at 3 dpf. Since the severity of the phenotype increases with age, it is conceivable that defects in type 1 collagen and elastin occur later in *atp6v1e1b^hi577aTg/hi577aTg^* compared to *atp6v1e1b^cmg78/cmg78^*.

**Reviewer #3**

The manuscript “Loss of a subunit of vacuolar ATPase identifies unexpected biological signatures of reduced organelle acidification in vivo” by Lore P. et al. describes the various phenotypes of zebrafish atp6v1e1b KO mutant. The authors showed lots of data (transcriptome and metabolome) and I think that the manuscript seems to be interesting as it identified the roles of vacuolar ATPase function, but to be quite descriptive. Although the authors expressed their results as “unexpected biological signatures” in the title, almost all results in this manuscript are the “expected biological signatures” from the previous results in other model systems and human diseases. I think that the most important point in this manuscript is connection between organelle acidification through the vATPase and mitochondrial respiration. Are these two phenomena (organelle acidification by vATPase and mitochondrial respiration) tightly linked each other or is the mitochondrial respiration just side effect? I think the direct connection between them in cellular & molecular levels (some mechanistic analyses) is needed for publication in PLOS Genetics.

We appreciate the comments of the reviewer and agree that the link between v-ATPase and mitochondrial function is a key finding in our study. It is known from previous *in vitro* studies that Bafilomycin A1 (which interacts with the V0 ring of v-ATPase, inhibiting proton translocation), saliphenylhalamide (locks v-ATPase in an assembled state) and chloroquine (which does not inhibit v-ATPases but disrupts lysosomal acidification) can all completely shut down mitochondrial oxygen consumption in mouse embryonic fibroblasts. To address this direct link between organelle acidification by v-ATPase and mitochondrial respiration *in vivo*, we have performed additional experiments in zebrafish (S9 Fig.). We show that Bafilomycin A1 acutely reduces the OCR in WT controls but does not completely block the oxygen consumption *in vivo*, similar to the effect of genetic *atp6v1e1b* deficiency. Interestingly, some WT controls treated with Bafilomycin A1 also show an increase in OCR after administration of oligomycin, again recapitulating the observations in *atp6v1e1b*-deficient zebrafish. It is possible that biological variability in other WT controls give mixed responses to administration of oligomycin after Bafilomycin A1 treatment. Moreover, Bafilomycin A1 also influences the basal OCR of *atp6v1e1b^hi577aTg/hi577aTg^* but not of *atp6v1e1b^cmg78/cmg78^*, likely due to the presence of residual atp6v1e1 protein expression. Taken together, these data strongly argue for a direct link between v-ATPase function and mitochondrial respiration.

Yambire KF, Rostosky C, Watanabe T, Pacheu-Grau D, Torres-Odio S, Sanchez-Guerrero A, et al. Impaired lysosomal acidification triggers iron deficiency and inflammation in vivo. eLife. 2019;8. Epub 2019/12/04. doi: 10.7554/eLife.51031.

A minor point
In this manuscript, the authors mentioned that gene expression profile (HIF1a, Notch, Wnt, and mTOR signaling) in zebrafish atp6v1e1b KO mutant is different from that in in vitro systems. But there are two atp6v1e1 genes, atp6v1e1a and atp6v1e1b in zebrafish. Thus, they should discuss about this point.

We agree with the reviewer that it is important to include more discussion regarding the role of *atp6v1e1a* in our manuscript. We have now added new data showing whole mount in situ hybridization to assess the tissue distribution of *atp6v1e1a* and *atp6v1e1b* expression in the revised manuscript, showing a similar spatial and temporal expression pattern of these orthologs in zebrafish (S1 Fig.). In addition, we have also generated a knockout zebrafish model for *atp6v1e1a*. This mutant model survives until adulthood and does not show any pathological features. Together with our new data showing that total atp6v1e1 protein levels are almost completely depleted in *atp6v1e1b*-deficient zebrafish (S2 Fig.), our results indicate that *atp6v1e1b* is the predominant ortholog in zebrafish. We have now included data on this mutant model in the revised manuscript to support the hypothesis that *atp6v1e1a* is a non-functional ohnolog with likely no or only very limited physiological relevance (S1 Fig.).

**Reviewer#4**:

In this work, the authors use mutant zebrafish larvae to investigate the physiological and cellular impacts of loss of function of the Atp6v1e1b subunit of the V-ATPase complex that pumps protons against concentration gradients. Interestingly, atp6v1e1b-/- mutant zebrafish develop several phenotypes—including craniofacial defects, cardiovascular defects, epidermal/skin defects, and protein glycosylation defects—that are reminiscent of problems observed in human patients with cutis laxa caused by mutations in ATP6V1E. The characterization and quantification of these phenotypes in zebrafish larvae, using two distinct atp6v1e1b-/- mutant lines, is comprehensive and convincing. These results suggest atp6v1e1b-/- mutant zebrafish provide a useful new animal model to study mechanisms underlying cutis laxa. The authors go on to use omics approaches to assess global changes in transcription, metabolism, and lipids in atp6v1e1b-/- mutants. These results give a broad overview of changes that occur in the mutants, and suggest several pathways/processes that are altered. A pharmacological approach is used to test whether targeting some of these processes could ameliorate phenotypes, but upon observing no positive effects, the authors conclude that targeting a single pathway is not sufficient to improve defects induced by loss of Atp6v1e1b. Overall, this work contributes to our understanding of in vivo consequences of altered V-ATPase function, but limitations of the global approach (pointed out below) appear to have prevented the authors from using their omics datasets to identify mechanisms that underlie specific phenotypes seen in mutant zebrafish (and human patients). Specific points are outlined below.

Major points:
1) There are a few caveats to using zebrafish genetics that should be clarified. One is that there are two zebrafish genes that encode for the V1E subunit-- atp6v1e1a and atp6v1e1b. It is not clear what cell types express either of these genes, and whether expression is overlapping (which may suggest functional redundancy) or cell-type specific. Clearly there are significant phenotypes and lethality in atp6v1e1b-/- mutants, but the atp6v1e1a gene should be discussed.

We thank the reviewer for this comment and acknowledge the importance of including and discussing the role of *atp6v1e1a* in our manuscript. To address this question, we have performed whole mount in situ hybridization to assess the tissue distribution of *atp6v1e1a* and *atp6v1e1b* expression. We observed similar spatial and temporal expression pattern of these orthologs in zebrafish (S1 Fig.). We have now included these data in the revised manuscript. Our new immunoblot analysis data have also shown that combined atp6v1e1 protein levels are all but eliminated in *atp6v1e1b*-deficient zebrafish larvae. Finally, we confirmed that loss of *atp6v1e1a* in zebrafish does not induce any pathological features nor early mortality. indicating that *atp6v1e1b* is the predominant ortholog in zebrafish. We have now included extra information on this mutant KO model as evidence of the hypothesis that *atp6v1e1a* is a non-functional ohnolog with likely no or only very limited physiological relevance (S1 Fig.).

2) A second caveat is that V-ATPase subunits are maternally supplied (mRNA and possibly protein). This maternal supply is likely the reason why mutant embryos can develop relatively normally for the first couple of days and survive up to 8 days. The gradual onset of phenotypes in mutants suggests a gradual loss of maternal supply. Thus, it is not clear how much of the maternal Atp6v1e1b protein supply is depleted at 3 dpf when the global omics studies were done. This should be discussed.

We agree with the reviewer that we cannot completely exclude potential contributions of maternal mRNA to the results obtained in our analyses. Unfortunately, since the *atp6v1e1b*-deficient zebrafish are not viable, maternal zygotic knockout embryos cannot be generated. Therefore, the only way to address this issue in some way would be to perform a deyolking procedure prior to sample collection in order to remove most maternally-derived mRNA and protein contributions. Nevertheless, this procedure cannot revert previous maternal effects during development, and might also cause disruption of other biological processes by itself. Therefore, we decided to investigate the intact embryos for the majority of the omics analysis to cause the minimal amount of artefactual disturbances to the measurements performed in this study. We took notice of this issue in the discussion.

The relative contribution of the maternal and paternal transcriptome in developing zebrafish embryos has previously been investigated using RNAseq analysis. These data indicated that *atp6v1e1b* mRNA was almost exclusively maternally-derived up to 6 hpf. Starting at 9 hpf however, tan equal contribution of paternal and maternal *atp6v1e1b* transcripts was detected, indicating a strong induction of zygotic expression. These data suggest that maternally-derived *atp6v1e1b* transcripts only play a role during early embryogenesis, and that zygotic *atp6v1e1b* transcription takes over after gastrulation. This time point corresponds to activation of the zygotic genome during the maternal to zygotic transition which normally takes place around the midblastula transition. Considering the time point of our analyses (2-4 dpf), we expect only very minimal residual impact of maternally-derived *atp6v1e1b* transcript and protein.

Rahlouni F, Szarka S, Shulaev V, Prokai L. A Survey of the Impact of Deyolking on Biological Processes Covered by Shotgun Proteomic Analyses of Zebrafish Embryos. Zebrafish. 2015;12(6):398-407. doi:10.1089/zeb.2015.1121

Harvey SA, Sealy I, Kettleborough R, Fenyes F, White R, Stemple D, et al. Identification of the zebrafish maternal and paternal transcriptomes. Development (Cambridge, England). 2013;140(13):2703-10. Epub 2013/05/31. doi: 10.1242/dev.095091. PubMed PMID: 23720042; PubMed Central PMCID: PMCPMC3678340.

3) A limitation to this study is that whole larvae were used to assess changes transcription, metabolism, lipids, and glycosylation. While V-ATPase probably has global cellular ‘house keeping’ functions, it is also likely that cell-type specific functions regulate processes such as Fe availability, hypoxia response, and signaling (mTOR, Notch, Wnt, etc) that underlie specific phenotypes. The authors touch on this in the Discussion, but this point could be expanded. Future work using cell-type specific approaches may uncover differences in mutants that shed light on mechanisms underlying mutant phenotypes in a given cell type.

We agree with the reviewer that one of the major limitations of the current study is the fact that we used whole embryos and larvae deficient for *atp6v1e1b* in all cell types for our experiments. Future studies will focus on investigating the biological response of tissue-specific and/or inducible inactivation of *atp6v1e1b* in zebrafish using the UAS-Gal4 system for tissue-specific expression of Cas9 protein or using vector-based approach to drive Cas9 expression in a tissue-restricted manner under specific promotors. This approach will likely enable us to also circumvent the early lethality observed in the currently used mutant models, allowing us to investigate the impact of *atp6v1e1b* during other stages beyond larval development. The availability of older zebrafish with disrupted atp6v1e1b function will also facilitate the study of tissue- and cell-type specific experimental approaches.

Minor points:
1) For the retroviral insertion allele, I would predict the insertion in the 5’ UTR would interfere with mRNA transcription, and not induce NMD as stated. This should be clarified.

We agree with the reviewer that the insertion in the 5’UTR interferes with the mRNA transcription rather than induce NMD. We have now included new data which shows that, in line with the decreased gene expression, atp6v1e1 protein expression is significantly reduced in *atp6v1e1b^hi577aTg/hi577aTg^* zebrafish (S2 Fig.). We have also adapted the statement regarding the effect of the 5’ UTR retroviral insertion on mRNA transcription in the revised manuscript.

2) In Sup Fig 1C, is this dpf or hpf? The text indicates mutants only survive 8 days.

We thank the reviewer for notifying us of this mistake. In the revised manuscript, we have changed it to hpf.

3) Pericardial edema in Fig 1A should be pointed out with an arrow.

In the revised manuscript we have pointed out the pericardial edema with an arrow.

4) For most analyses, the two atp6v1e1b-/- mutants have identical phenotypes. There are two exceptions reported: blood flow (Fig 1G) and complex N-glycans (Fig 2G) were altered in one mutant but not the other. It would be useful to comment on differences between the mutants.

In the revised manuscript, we have elaborated on the differences between both mutant zebrafish models in the discussion.

5) With regard to the possibility of specific spatial and temporal functions for atp6v1e1b (see major points 1-3 above), it should be clarified what cell type and what stage of development we are looking at in Sup Fig 2G.

In the revised manuscript, we have added a schematic presentation of the area scanned by confocal microscopy in S3 Fig.G to clarify the spatial distribution, showing the presence of acidified vesicles in the brain region. Furthermore, we have also included a quantification of the acidified vesicles (endosomes, lysosomes, exosomes, ...) in S3 Fig.H. The developmental stage (3 dpf) is also clearly indicated in the revised figure legend.
