# Supplementary material for: Loss of zebrafish atp6v1e1b, encoding a subunit of vacuolar ATPase, recapitulates human ARCL type 2C syndrome and identifies multiple pathobiological signatures
Source: PLoS Genet. 2021 Jun 18;17(6):e1009603. doi: 10.1371/journal.pgen.1009603 (PMC8244898; doi:10.1371/journal.pgen.1009603)
Supplement: Attachment — Submitted filename: response to reviewers_second revision round_09042021.DOCX [file pgen.1009603.s017.DOCX]

Responses to Reviewers’ Questions

Comments to the Authors:

Please note here if the review is uploaded as an attachment. The review will be uploaded as an attachment in the editorial manager.

Reviewer #1: Please find enclosed the comments to the research article “Loss of zebrafish atp6v1e1b, encoding a subunit of vacuolar ATPase, recapitulates human ARCL type 2 syndrome and identifies multiple pathobiological signatures of reduced vesicular acidification, PGENETICS-D-20-01563_R1” submitted for publication in PLOS Genetics by Bert Callewaert and colleagues. This is article has been strongly revised by the authors and all comments from the reviewer were carefully addressed. I recommend publication with a minor revision.

We thank the reviewer for positively reviewing our revised manuscript and for the extra suggestions.

Some minor points:

The authors use the term type 2 cutis laxa syndrome frequently. For example within the title and the abstract they state: “…recapitulates human ARCL type 2 syndrome “ and “…cause type 2 cutis laxa, a condition further characterized by…”. According to OMIM there are different forms of type 2 cutis laxa. I suggest within the title they should use the term ARCL type 2 C syndrome and within the abstract the plural; a group of conditions. Please carefully check the remaining manuscript for this point.

We have changed our title to ‘Loss of zebrafish *atp6v1e1b*, encoding a subunit of vacuolar ATPase, recapitulates human ARCL type 2C syndrome and identifies multiple pathobiological signatures’ and cross-referenced our manuscript for the correct description of the cutis laxa syndrome subtypes.

Manuscript with text changes:

Line 292: The authors state the golgi is more frequently seen within the WT. This to me is rather due to the sectional plane and due to the fact that the golgi is more fragmented within the mutants and therefore not frequently seen within the mutant sections. This should be clarified.

We agree with the reviewer that the reduced number of Golgi apparatus observed in *atp6v1e1b*-deficient larvae might be due to the sectional plane imaging and the fragmentated nature of this phenotype. We have amended our previous statement in the revised manuscript on line 243-245.

Figure S2: The atp6v1e1 protein quantification shows that in the fish with the proviral insert the protein amount is relatively strong in comparison to the CRISPAR mediated KO. In one lane from the later line a similar protein amount is detectable whereas the two other lanes show a complete absence of the protein, as expected. Can the authors state on this point?

We have repeated the immunoblot multiple times in order to address this question. We consistently observe higher levels of atp6v1e1 protein in the samples from zebrafish with the proviral insert compared to the CRISPR/Cas9-induced KO samples, indicating some residual protein expression in the former line as noted in the manuscript. On the other hand, we have repeatedly observed a very low level of atp6v1e1 protein in one of the CRISPR-mediated *atp6v1e1b*-deficient samples. Considering the sequence homology with the human ATP6V1E1 protein fragment which was used as an immunogen to generate the antibody, we expect that both zebrafish atp6v1e1a and atp6v1e1b proteins are recognized on Western blot. Based on the fact that the CRISPR-induced indel mutation causes a frameshift, we do not expect that full-length atp6v1e1b protein can be generated from this allele. Therefore, we conclude that the remaining signal in the CRISPR-induced *atp6v1e1b* KO sample originates from atp6v1e1a protein. We have added these sentences in the revised manuscript on line 160-164.

Line 343: The authors state: “…To our surprise, atp6v1e1b-deficient zebrafish displayed similar total p70-S6K1 protein expression and phosphorylation levels as observed in WT controls…” To me it looks like there might be changes, however they are minor. I think the interpretation might be taken because the two control lines are not congruent. I suggest clarification within the results section.

We have clarified this section in more details. We observed a trend of decreased p70-S6K1 protein levels in the *atp6v1e1b*-deficient zebrafish which did not reach statistical significance (p=0858 for *atp6v1e1b*^hi577aTg/hi577aTg^ and p=0.0879 for *atp6v1e1b*^cmg78/cmg78^). Due to the large variability of phospho-p70-S6K protein levels within and between WT control lines, the ratio of phospho-p70-S6K/ p70-S6K protein level was not statistically different (p=0,7520 for *atp6v1e1b*^hi577aTg/hi577aTg^ and p=0.1909 for *atp6v1e1b*^cmg78/cmg78^). In addition, we have noticed a small inconsistency in the data-analysis which we corrected in the revised manuscript (Fig3D-E). We have adapted the sentence on line 300-304: “A trend was noted toward decreased levels of p70-S6K1 protein levels in both *atp6v1e1b*-deficient zebrafish, which did not reach statistical significance. Due to the large variability of phospho-p70-S6K protein levels within and between WT control lines, the ratio of phospho-p70-S6K/ p70-S6K protein level remained stable between the different conditions.”.

Reviewer #2: The authors have answered most of my questions. Thank you very much for the Discussion points!

However, since the authors do recognize pH measurement isn’t straightforward in zebrafish, and could well be ascertained in future studies, I suggest softening the conclusion on acidification based on their current results, and at least mentioning the caveats on the Discussion.

We appreciate the reviewer’s approval of all our amendments in the previous revised manuscript. We acknowledge that reduced acidification was only shown by Lysotracker Red. We have softened the conclusion throughout the results, abstract and title but particularly on line 289 and we have mentioned that additional experiments are needed to confirm that loss of *atp6v1e1b* leads to increased pH in the acidic vesicles in the discussion on line 502-505.

Reviewer #3: The authors answered all my questions. I recommend that their manuscript should be published in Plos Genetics.

Thank you for the positive feedback!

Reviewer #4: The authors have made substantial revisions that have largely addressed my previous comments and significantly improved the manuscript. However, this paper remains descriptive, and does not provide new mechanistic insight into any of the many phenotypes described. In addition, there are still parts of the paper that are confusing and require additional clarification.

Minor points:

1) In the first section of the Results (starting on line 136), the back-and-forth flow between Fig. S1 and Fig. S2 is confusing and difficult to follow. The text describes atp6v1e1b knockout first and then atp6v1e1a, whereas this is in the reverse order in the figures (Fig. S1= atp6v1e1a and Fig. S2= atp6v1e1b). I suggest making the description of these mutants easier to follow.

We agree with the reviewer that the flow of the manuscript would benefit from a more natural order of description of the results obtained in the zebrafish mutants. In the revised manuscript, we have first discussed the molecular features of the *atp6v1e1a* mutant zebrafish model on line 145, followed by the results of the molecular analysis and preliminary phenotype of the *atp6v1e1b* mutant.

2) In Fig. S2D-E western blots, it is not described what is loaded in the three lanes for wt and mutants. It should be clarified is these are the same extracts or different extracts.

We acknowledge the reviewer’s suggestion. We have added the sentence: “Different protein extracts were loaded on the immunoblot.”. However, we have mentioned in the figure legend that the data are expressed as mean ± SD from 3 biological replicates. We used 3 biological replicates in which we pooled 20 zebrafish larvae in order to obtain the corresponding lysate.

3) For Fig. S5I, is this dpf or hpf?

We thank the reviewer for notifying us of this mistake. In the revised manuscript, we have changed it to hpf.

4) The data and conclusions regarding HIF-1 is confusing. The authors state (starting in line 290) “HIF-1a-target genes (egln3, vegfaa, 291 vegfab, slc2a1a, slc2a1b, pfkfb3, angptl4 and pdk1) were downregulated in atp6v1e1b-deficient zebrafish at 3 dpf (S5 Fig.A-H)” and then conclude “Taken together, we show that loss of atp6v1e1b does not alter the Notch and HIF-1a signaling pathways…” It should be clarified why the authors conclude that HIF-1a signaling is not affected.

We thank the reviewer for this comment and acknowledge that the description of these results and the corresponding conclusion was not entirely clear. Previous *in vitro* work has highlighted that decreased endo(lyso)somal acidification leads to reduced cytosolic bioavailability of iron and subsequent induction of HIF-1α signaling. Based on these results, we originally hypothesized to observe an increase in the HIF-1α-target genes (*egln3*, *vegfaa*, *vegfab*, *slc2a1a*, *slc2a1b*, *pfkfb3*, *angptl4* and *pdk1*) in the *atp6v1e1b*-deficient zebrafish. Contradictory, our results showed a downregulation of these target genes, concluding that HIF-1α signaling is not induced by decreased endo(lys)somal acidification *in vivo*. As correctly noticed by the reviewer, this does not take away the fact that the HIF-1α-signaling pathway is indeed affected, albeit in the opposite direction as previously shown in *in vitro* experiments. We have rephrased our conclusion on line 320 to “Taken together, we show that loss of *atp6v1e1b* *in vivo* does not stimulate the HIF-1α signaling pathways (in contrast to previous *in vitro* work) and does not alter the Notch signaling pathway, but does have an impact on mTOR, AMPK and Wnt signaling.”.

5) In line 448 the authors conclude “While zebrafish deficient in atp6v1e1a survived normally to adulthood without any phenotype, suggesting that this gene is a non-functional ohnolog…” I suggest softening the statement that atp6v1e1a is ‘non-functional,’ since there may be unrecognized compensatory mechanisms that could be discovered in future work.

We have removed our statement “Hence, we conclude that *atp6v1e1a* is a non-functional ortholog of ATP6V1E1.” in the results section and amended our statement in the discussion on line 477-478.
